# RAD18 directs DNA double-strand break repair by homologous recombination to post-replicative chromatin

**DOI:** 10.1093/nar/gkae499

**Published:** 2024-06-13

**Authors:** Matous Palek, Natalie Palkova, consortium CZECANCA, Marta Cerna, Klara Horackova, Milena Hovhannisyan, Marketa Janatova, Sandra Jelinkova, Petr Nehasil, Jana Soukupova, Barbora Stastna, Petra Zemankova, Lenka Foretova, Eva Machackova, Vera Krutilkova, Spiros Tavandzis, Leona Cerna, Stepan Chvojka, Monika Koudova, Ondrej Havranek, Jan Novotny, Kamila Vesela, Michal Vocka, Lucie Hruskova, Renata Michalovska, Denisa Schwetzova, Zdenka Vlckova, Monika Cerna, Marketa Hejnalova, Nikol Jedlickova, Ivan Subrt, Tomas Zavoral, Marcela Kosarova, Gabriela Vacinova, Maria Janikova, Romana Kratochvilova, Vaclava Curtisova, Radek Vrtel, Ondrej Scheinost, Petra Duskova, Viktor Stranecky, Petra Kleiblova, Zdenek Kleibl, Libor Macurek

**Affiliations:** Cancer Cell Biology, Institute of Molecular Genetics of the Czech Academy of Sciences, Prague CZ-14220, Czech Republic; Department of Cell Biology, Faculty of Science, Charles University, Prague, Czech Republic; Cancer Cell Biology, Institute of Molecular Genetics of the Czech Academy of Sciences, Prague CZ-14220, Czech Republic; Department of Cell Biology, Faculty of Science, Charles University, Prague, Czech Republic; Cancer Cell Biology, Institute of Molecular Genetics of the Czech Academy of Sciences, Prague CZ-14220, Czech Republic; Institute of Medical Biochemistry and Laboratory Diagnostics, First Faculty of Medicine, Charles University and General University Hospital in Prague, Prague, Czech Republic; Institute of Medical Biochemistry and Laboratory Diagnostics, First Faculty of Medicine, Charles University and General University Hospital in Prague, Prague, Czech Republic; Institute of Medical Biochemistry and Laboratory Diagnostics, First Faculty of Medicine, Charles University and General University Hospital in Prague, Prague, Czech Republic; Cancer Cell Biology, Institute of Molecular Genetics of the Czech Academy of Sciences, Prague CZ-14220, Czech Republic

## Abstract

RAD18 is an E3 ubiquitin ligase that prevents replication fork collapse by promoting DNA translesion synthesis and template switching. Besides this classical role, RAD18 has been implicated in homologous recombination; however, this function is incompletely understood. Here, we show that RAD18 is recruited to DNA lesions by monoubiquitination of histone H2A at K15 and counteracts accumulation of 53BP1. Super-resolution microscopy revealed that RAD18 localizes to the proximity of DNA double strand breaks and limits the distribution of 53BP1 to the peripheral chromatin nanodomains. Whereas auto-ubiquitination of RAD18 mediated by RAD6 inhibits its recruitment to DNA breaks, interaction with SLF1 promotes RAD18 accumulation at DNA breaks in the post-replicative chromatin by recognition of histone H4K20me0. Surprisingly, suppression of 53BP1 function by RAD18 is not involved in homologous recombination and rather leads to reduction of non-homologous end joining. Instead, we provide evidence that RAD18 promotes HR repair by recruiting the SMC5/6 complex to DNA breaks. Finally, we identified several new loss-of-function mutations in *RAD18* in cancer patients suggesting that RAD18 could be involved in cancer development.

## Graphical Abstract

**Graphical Abstract:**
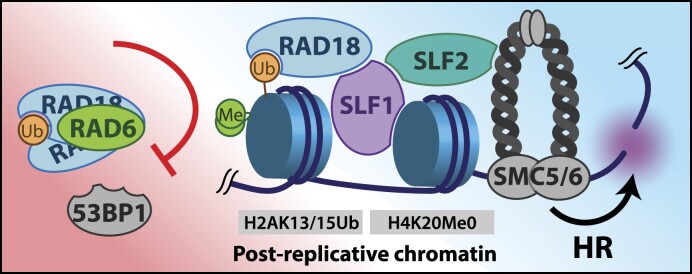

## Introduction

Genome instability is a driver of tumorigenesis and therefore integrity of the genome needs to be actively protected by various DNA repair and cell cycle checkpoint mechanisms to prevent accumulation of potentially harmful mutations (1,2). DNA double strand breaks (DSBs) are repaired by two main pathways, non-homologous end joining (NHEJ) and homologous recombination (HR) that differ in the fidelity of repair, cell cycle dependencies and repair dynamics. Whereas the rapid and error-prone NHEJ can operate throughout the cell cycle, the more precise HR is restricted to S/G2 phases of the cell cycle (3). HR depends on resection of the DNA and invasion of the single stranded DNA into the helix of the sister chromatid that serves as the template for DNA synthesis. Breast cancer type 1 susceptibility protein (BRCA1) mediates multiple steps of HR including DNA resection, RAD51 filament formation, DNA strand invasion and DNA resolution (4). In contrast, recruitment of TP53-binding protein 1 (53BP1) and its associated protein complexes RIF1 and shieldin suppresses DNA resection and thus blocks DNA repair by HR (5). DNA repair choice is thus largely mediated by a balance between BRCA1 and 53BP1 activity at DNA lesions (6). Recruitment of both 53BP1 and BRCA1 depends on ubiquitination of the histone H2A at K13/15 by RNF168 (7,8). In addition, tandem Tudor domains of 53BP1 bind H4K20me2 that is broadly distributed in matured chromatin and its dilution in post-replicative chromatin can decrease binding of 53BP1 to DSBs in the S/G2 phase of the cell cycle (9). In contrast, BRCA1 is recruited to DSB sites by its partner BARD1 that utilizes the ankyrin repeat domain (ARD) to recognize non-methylated histones incorporated into the newly replicated chromatin during S phase (10). Similarly, TONSL–MMS22L complex that promotes RAD51 loading is recruited by ARD of TONSL that reads the non-methylated histone H4K20me0 in the post-replicative chromatin (11,12). Genetically determined defects in HR pathways promote development of cancer and also influence sensitivity of cancer cells to chemotherapy including the PARP inhibitors (13–16).

Collapse of the stalled replication forks is considered a major threat for genome integrity. Translesion DNA synthesis (TLS) enables replication of a damaged DNA template and thus prevents replication fork stalling. TLS is mediated by E3 ubiquitin ligase RAD18 that interacts with E2 conjugating enzyme RAD6 (encoded by *UBE2A* and *UBE2B* genes) and monoubiquitinates Proliferating cell nuclear antigen (PCNA) mediating recruitment of Y-family DNA polymerases. Due to the low fidelity of translesion DNA polymerases, TLS is error-prone and can lead to accumulation of mutations in cancer cells (17). Indeed, expression of RAD18 has been linked with accumulation of the carcinogen-induced TLS-dependent A > T and G > T single nucleotide variations (18). On the other hand, loss of RAD18 in tumors caused accumulation of indels resembling a mutation signature of the BRCA1-deficient breast cancer and suggesting that RAD18 plays also TLS-independent role during cancerogenesis (18). DNA repair reporter assays based on induction of nuclease-induced DSBs showed that RAD18 promotes HR repair (19,20). Moreover, overexpression of RAD18 was reported to enhance homology-directed repair by disruption of 53BP1 nuclear foci (21,22). This observation was supported by *in vitro* assays that showed a significantly higher affinity of the ubiquitin binding UBZ domain of RAD18 to H2A-K15Ub compared to low affinity observed for 53BP1 (23). On the other hand, RAD18 has also been reported to ubiquitinate 53BP1 and to promote its retention on chromatin in G1 cells (24). Therefore, the physiological role of endogenous RAD18 in displacement of 53BP1 from DSBs and its function in DNA repair are currently unclear.

In response to different DNA damage modalities, RAD18 forms various complexes. After UV-induced damage, RAD18–RAD6 complex first monoubiquitinates PCNA, then RAD18 interacts with helicase-like transcription factor (HLTF), promoting PCNA polyubiquitination and bypass of the DNA lesion by template switching. Alkylating agent MMS triggers RAD18 interaction with SNF2 histone linker plant homeodomain RING helicase (SHPRH) enabling precise TS bypass (25). Filling of ssDNA gaps that arise during DNA replication is mediated by RAD18–REV1–Polζ complex and becomes particularly important in BRCA-deficient tumors (26,27). Finally, RAD18 association with the SLF1–SLF2–SMC5/6 complex was implicated in DNA inter-strand crosslink repair and break-induced replication-like DNA synthesis at stalled replication forks upon ectopic RNF168 overexpression (28,29). Molecular basis of the protein complexes of RAD18 that contribute to the regulation of DSB repair remain largely unexplored.

Here, we investigated the function of RAD18 in DSB repair in human cells. Using RAD18–KO cells we show that endogenous RAD18 is required for counteracting 53BP1 recruitment to DNA lesions and this activity can be rescued by expression of the wild-type but not the UBZ-deletion RAD18 mutant. Next, we confirm that this RAD18 function is dependent on specific recognition of histone H2A ubiquitinated at K13/15 by RNF168. We used STED nanoscopy to investigate the topology of repair foci and found that RAD18 accumulates at 2–3 chromatin nanodomains close to the BRCA1 resection center of the IR-induced foci, whereas 53BP1 is distributed at the surrounding chromatin. We also performed functional analysis of a panel of cancer-related *RAD18* variants and identified several mutations with abrogated IR-induced foci accumulation and 53BP1 -inhibitory activity. Next, we identified K186, K197, K201 and K218 as major auto-ubiquitination sites of RAD18 that diminish its accumulation at damaged chromatin. Further, we show that RAD18 accumulates at DSBs predominantly in S/G2 phases in a manner dependent on recognition of H4K20me0 by the ARD domain of SLF1. Finally, we demonstrate that restriction of 53BP1 by endogenous RAD18 reduces NHEJ but surprisingly does not affect HR efficiency. Instead, we present a model where RAD18 promotes HR repair by recruiting the SMC5/6 complex specifically to DSB sites.

## Materials and methods

### Cells

Human hTERT-immortalized RPE1 cells (referred to as RPE), human osteosarcoma U2OS cells, and HEK293 cells were grown in high-glucose DMEM supplemented with 6% FBS (Gibco), Penicillin (10 U/ml) and Streptomycin (0.1 mg/ml). All cell lines were regularly tested for mycoplasma contamination using MycoAlert kit (Lonza). U2OS–RAD18–KO cells were generated by transfection of U2OS cells with a pool of plasmids coding for Cas9, three different sgRNAs targeting RAD18 and a selection cassette containing RFP and puromycin resistance genes flanked by the homology arms (Santa Cruz, sc-425476, sc-425476-HDR). After selection with puromycin, the RFP positive cells were sorted using FACS. Finally, the selection cassette was removed by infection with Cre Recombinase Adenovirus (Vector Biolabs). To generate RPE–RAD18–KO cells, parental RPE cells were co-transfected with 70 nM sgRNA (GAGUGAAUUUCAGCUUCUGG, Synthego) targeting the exon 10 of *RAD18* with purified TrueCut protein Cas9 v2 using CRISPRMAX (both Thermo Fisher Scientific) and single cell clones were expanded. Following the same procedure, RPE-53BP1-KO cells were generated using sgRNA (ACCCTCAAGGAACACTCCA) targeting exon 17 of *TP53BP1*. RPE-iCut-RAD18_KO cells were generated by induction of Cas9 expression by overnight treatment with 2 μM doxycycline and 1 μM Shield-1 (Aobious) followed by transfection of sgRNA. The inactivation of *RAD18* or *53BP1* gene was confirmed by western blotting and sequencing. The traffic light reporter U2OS-TLR cells were generated and validated previously (30). To induce Cas9 expression, RPE-iCut cells were treated overnight with doxycycline and Shield-1. Formation of discrete DSBs was induced by transfection of 45S rDNA sgRNA (GCGGTGCGTGACGGGCGAGG) a mixture of 12 single cutter sgRNAs (SSC_6171, SSC_3199, SSC_3344, SSC_3718, SSC_4131, SSC_4247, SSC_0582, SSC_0959, SSC_1042, SSC_2059, SSC_1357, SSC_1604) at final concentration 2.5 nM using Lipofectamine RNAiMAX (Thermo Scientific) (31). Silencer Select siRNAs (Thermo Scientific) were transfected using RNAiMAX at final concentration 5 nM. Where indicated, siRNA transfection was repeated after 2 days to improve depletion efficiency. Finally, cells were harvested after 2 days from the second transfection. Following siRNAs were used and validated in this study: RAD18 (GUUCAGACAUCAUAAGAGA), SETD8 (GAAUCUACAGGAAACGAGA), UBE2A (CCAGGAGAAC-AAACGGGAA), UBE2B (CCAAAAUGUUUCAUCCAAA), RNF168 (GAGUAUCACUUACGCG-CUA), RNF8 (GGAG-AAUGCGGAGUAUGAA), SMC5 (GGUUGAUUGCCUUACGUGA), SLF1#1 (CCACCAGUGUUCAUACUGA), SLF1#2 (GAACCUUACUAAUGCUGAA) and BRCA1 (GGGAUACCAUGCAACAUAA). The siRNA targeting TP53BP1 (GAAGGACGGAGUACUAAUA) was validated previously (30). Transfection of plasmid DNA was performed using polyethylenimine in the ratio 1:6. Where indicated, cells were IR irradiated using X-RAD 225XL (Precision X-Ray) with 2 mm aluminium filter. If not stated otherwise a dose 3 Gy was used. For UV-irradiation, cells grown on culture plates were PBS washed and irradiated using UV Crosslinker CL-1000 (UVP) before addition of fresh medium. If not stated otherwise a dose 20 J/m^2^ was used.

### Antibodies and reagents

The following antibodies were used in this study: BRCA1 (clone D-9, sc-6954, 1:100 for IF), RAD6 (UBE2A, clone G-9, sc-365507, 1:1000 for WB), Ubiquitin (clone P4D1, sc-8017, 1:500 for WB), PCNA (clone PC10, sc-56, 1:1000 for WB) from Santa Cruz; FLAG (clone M2, F 1804, 1:300 for IF), γH2AX (clone JBW301, 05–636, 1:1000 for WB), 53BP1 (clone BP-13, MAB3802, 1:300 for IF), GFP (clones 7.1 and 13.1, #11814460001, 1:1000 for WB), BrdU (clone BU-33, B8434, 1:100 for IF), anti-conjugated ubiquitin (FK2, 04-263, 1:2000 for IF) from Merck; γH2AX (clone D7T2V, #80312, 1:300 for IF), γH2AX (clone 20E3, #9718, 1:300 for IF), RAD18 (clone D2B9, #9040, 1:400 for IF, 1:1000 for WB), PCNA ubiquityl-Lys164 (clone D5C7P, #13439, 1:100 for IF), H2A (clone D603A, #12349, 1:1000 for WB) from Cell Signaling Technology; 53BP1 (NB100-305, 1:400 for IF), SMC5 (NB100-469, 1:300 for IF, 1:1000 for WB) from Novus Biologicals; RAD51 (ab176458, 1:400 for IF) and H4K20Me0 (ab227804, 1:2000 for IF, 1:1000 for WB) from Abcam. PageRuler Prestained Protein Ladder (10–180 kDa) was from Thermo Scientific.

### Plasmids

The sequence coding for the human RAD18 was cloned from pEGFP-N3-hRAD18 (Addgene ID: 68824, (32)) into pEGFP-C1 plasmid. Individual RAD18 variants were prepared by ligation of PCR fragments using Gibson assembly kit (NEB). The ΔUBZ and ΔR6BD RAD18 mutants were prepared by deletion of residues 201–224 and 340–380, respectively. The pCMV6-UBE2A-Myc-FLAG plasmid coding for the human orthologue of yeast RAD6 was obtained from Origene (RC204194). To facilitate visualization in cells, RAD6 was C-terminally tagged with EGFP by cloning into pEGFP-N3 plasmid. The pEGFP-C1-SLF1 plasmid was described previously (33). The fragments coding for residues 1–435 and 1055–1152 were deleted to clone the ΔBRCT and ΔARD SLF1 mutants, respectively. The pcDNA3-FLAG-RNF168 plasmid was a kind gift from Daniel Durocher (Addgene ID: 133976, (34)).

### High-content microscopy

Cells were grown on coverslips, PBS washed and fixed with 4% PFA for 15 min at RT. For Ub-PCNA and γTubulin staining, cells were fixed with methanol for 15 min at –20°C. For native BrdU staining, cells were treated with 30 μM BrdU for 48 h before IR-irradiation. Where indicated, cells were pre-extracted prior fixation in 25 mM HEPES pH 7.4, 50 mM NaCl, 1 mM EDTA, MgCl_2_, 300 mM sucrose, 0.5% Triton X-100 for 5 min. Upon permeabilization with 0.5% Triton-X100 in PBS for 5 min, cells were blocked in 1% BSA for 30 min. To distinguish individual cell cycle phases, cells were pulse labeled with EdU 30 min prior fixation or irradiation. Incorporated EdU was labelled using Click-iT reaction in 0.1 M Tris pH 8.5, 0.1 M sodium ascorbate, 2 mM CuSO_4_ and 10 μM AlexaFluor 647 azide (Thermo Scientific) for 30 min at RT. Coverslips were then incubated in primary antibodies for 2 h at RT, PBS washed, and incubated in secondary antibodies diluted 1:500 in 1% BSA for 1 h at RT. After incubation with 1 μg/ml DAPI for 2 min, coverslips were washed with H_2_O and mounted using Vectashield. Images were acquired using Olympus ScanR equipped with UPLXAPO 60×/1.42 OIL and UPLXAPO 40x/0.95 DRY CORR objectives. Olympus ScanR analysis software was used for image analysis and FlowJo (Tree Star) was used for data visualization.

### Dual-channel STED microscopy

Cells were seeded on 1.5H coverslips (Marienfeld) 1 day before irradiation. Coverslips were then fixed, permeabilized and blocked with BSA. After incubation with BRCA1 and RAD18/53BP1 primary antibodies, coverslips were incubated in secondary antibodies conjugated to STAR 580 or STAR 635P (Abberior). STED microscopy was performed using DMi8 with laser scanning confocal head Leica TCS SP8. Five optical sections were acquired using a HC PL APO 100×/1.4 OIL STED objective, oil n = 1.518, pinhole 1 AU, in a format 1504 × 1504 pixels with a voxel size 22 × 22 × 149.7 nm. Dyes STAR 580 and STAR 635P were sequentially excited with a white pulsed laser and depleted with pulsed 775 nm STED laser. Time-gated detection was set at 0.3–10 ns for both channels. Emitted photons were detected using HyD detector in a photon counting mode. To reduce noise, images were deconvolved using (Scientific Volume Imaging). For analysis of 53BP1/RAD18 distribution in DNA repair foci, only non-overlapping foci containing single central BRCA1 nanodomain were selected. Images were processed as maximum intensity projections. Positions of individual nanodomains were detected by Find Maxima function in ImageJ and their XY coordinates adjusted using plugin GaussFit_OnSpot to obtain sub-pixel resolution (https://imagej.nih.gov/ij/plugins/gauss-fit-spot/index.html). 53BP1 or RAD18 nanodomains distant >500 nm from the foci center, represented by BRCA1 nanodomain, were excluded from the analysis. Finally, 53BP1/RAD18 nanodomains count and their minimal Euclidean distance from the BRCA1 center were calculated using R.

### Traffic light reporter assay

U2OS cells stably expressing Traffic light reporter 1.1 (Addgene ID: 31482) were seeded to 6-well plate and reverse transfected with 5 nM siRNA. For co-depletion experiments, cells were reverse transfected next day with the second siRNA. 48 h after siRNA transfection, cells were co-transfected with plasmid pRRL sEF1a HA.NLS.Sce (opt).T2A.IFP (Addgene, ID:31484) and pRRL-SFFV-d20GFP.T2A.mTagBFP Donor plasmid (Addgene, ID:31485, (35)). To enhance depletion efficiency, cells were repeatedly transfected with 1 nM siRNA. After 72 h, fluorescence was measured using FACSymphony (BD Biosciences) and a fraction of GFP and RFP positive cells in a BFP positive cell population was analysed using FlowJo (TreeStar).

### Co-immunoprecipitation

Cells were lysed 24 h after transfection with lysis buffer composed of 50 mM Tris pH 8.0, 150 mM NaCl, 0.1% NP-40, 10% glycerol, 2 mM EDTA, 3 mM EGTA, 1 mM DTT and cOmplete EDTA-free Protease Inhibitor Cocktail. After sonication for 3 × 20 sec at 20% amplitude using Q125 Sonicator, samples were supplemented with 10 mM MgCl_2_, 100 U/ml benzonase and incubated for 30 min at ice. To ensure stripping of proteins from non-digested DNA, 50 μg/ml Ethidium Bromide was added, and the soluble fraction was obtained by centrifugation for 30 min at 4°C at 20 000 g. After incubation with GFP-Trap beads (Chromotek) at 4°C for 1 h, the beads were washed 5× with lysis buffer. Finally, immunoprecipitated proteins were eluted by incubation with SDS loading buffer for 5 min at 95°C and analyzed using SDS PAGE and immunoblotting.

### Acid histone extraction

HEK 293 cells were incubated in extraction buffer containing 0.5% Triton X100 in PBS, 2 mM PMSF, 0.02% NaN_3_, 30 mM *N*-ethylmaleimide for 10 min at 4°C. The insoluble fraction was collected by centrifugation at 800 g for 10 min. Pellet was washed with extraction buffer and incubated in 0.2N HCl at 4°C overnight. Next day, residual insoluble fraction was pelleted by centrifugation at 800 g for 10 min and discarded. The supernatant fraction was neutralized with 1 M Tris, pH 10 and snap frozen in liquid nitrogen.

### Histone and K63-linked polyubiquitin pull-down assay

EGFP–RAD18 was immunoprecipitated from transfected U2OS–RAD18–KO cells using GFP-Trap beads. The beads were then washed 2× with IP buffer containing 1 M NaCl, and 2× with IP buffer containing 150 mM NaCl. Next, the beads were incubated with 100 μl of chromatin extracts or 1.5 μg of PolyUb chains (Boston Biochem) for 2 h at 4°C in 500 μl binding buffer composed of 50 mM Tris pH 8.0, 150 mM NaCl, 0.1% NP-40, 10% glycerol, 2 μM ZnCl_2_, 0.1% BSA, 1 mM DTT. Finally, the beads were washed 2× with binding buffer and 3x with binding buffer without BSA. Pulled-down proteins were eluted by incubation with SDS loading buffer for 5 min at 95°C and analyzed using SDS PAGE and immunoblotting.

### Cell proliferation assay

Cells were seeded in 96-well plates at 100–200 cells per well. After 24 h, plates were irradiated as indicated and then incubated for 6 days. Finally, cells were incubated with 30 μg/ml resazurin (Sigma, R7017) for 3 h. Fluorescence intensity at 590 nm upon 560 nm excitation was measured using Envision plate reader (PerkinElmer).

### Cancer patients and controls

Genotype (presence of *RAD18* germline variants) and phenotype (cancer diagnoses) data were retrieved from the CZECANCA (Czech cancer panel for clinical application) consortium database. All enrolled individuals provided written informed consent approved by the Ethics Committees at the participating diagnostic centers prior germline genetic testing indicated by national testing guidelines. Germline genetic testing were conducted in accordance with the Declaration of Helsinki. All patients were analyzed by the CZECANCA panel (including *RAD18*) NGS workflow described in detail previously (36). Anonymized genotype data from the unselected population-matched controls were obtained from National Center for Medical Genomics (www.NCMG.cz). All patients and controls were of Czech European ancestry.

### Statistical analysis

Data visualization and statistical analysis was performed using Prism 5 (GraphPad Software). For quantitative microscopy data the two-tailed Mann–Whitney *U*-test was used for determination of statistical significance. Where indicated, the unpaired two-tailed T-test or the two-way ANOVA were used for data with assumed normal distribution. In all graphs, the central tendency of data is represented by the mean with error bars showing the standard deviation, ****P < 0.0001, ***P < 0.001, **P < 0.01, *P < 0.05. All experiments were reproduced with similar results at least two times. For quantitative microscopy, we evaluated >500 cells per condition for experiments where the whole population was analyzed, >150 cells per condition for experiments with cell cycle gating, and >125 cells per condition for experiments with transiently transfected cells. The association of germline *RAD18* pathogenic/likely pathogenic variants with the development of individual cancer types was tested using the two-sided Fisher's exact test with *P* < 0.05 considered statistically significant.

## Results

### RAD18 binds H2A ubiquitinated by RNF168 and negatively regulates 53BP1 at DSBs

**Figure 1.**
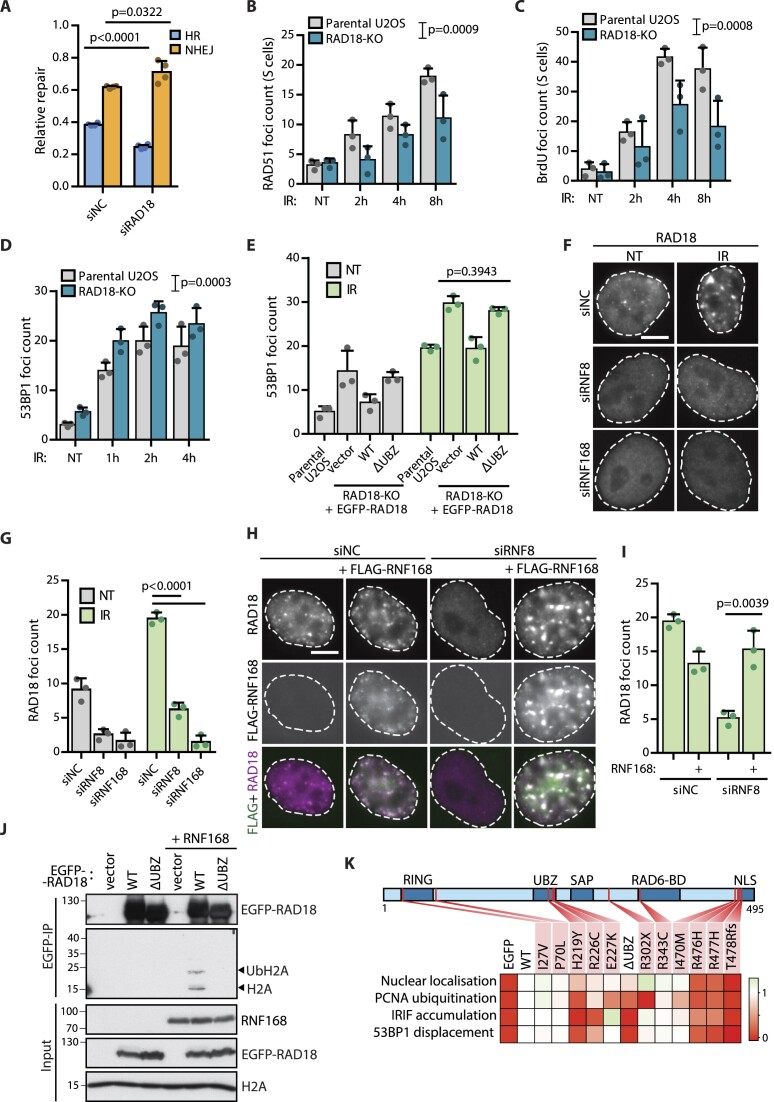
RAD18 negatively regulates 53BP1 at DSBs by recognizing H2A ubiquitinated by RNF168. (**A**) Relative NHEJ and HR repair in traffic light reporter U2OS cells treated with control (NC) or RAD18 siRNA. The repair efficiency was normalized to the total repair efficiency in the siNC treated cells (mean + SD, *n* = 4, two-tailed *t*-test). (**B**) High-content microscopy quantification of RAD51 foci in EdU positive U2OS parental or RAD18–KO cells upon IR-irradiation (mean + SD, *n* = 3, two-way ANOVA). (**C**) Quantification of ssDNA by native BrdU staining in U2OS parental or RAD18–KO cells irradiated with 10 Gy (mean + SD, *n*= 3, two-way ANOVA). (**D**) Quantification of 53BP1 foci in U2OS parental or RAD18–KO cells upon IR-irradiation (mean + SD, *n* = 3, two-way ANOVA). (**E**) Quantification of 53BP1 foci in U2OS parental cells and RAD18–KO cells transfected with wild-type EGFP–RAD18–WT, EGFP–RAD18–ΔUBZ or EGFP empty vector control. Where indicated, cells were IR-irradiated 2 h before fixation (mean + SD, *n* = 3, two-tailed *t*-test). (**F**) U2OS cells were treated with control, RNF8, or RNF168 siRNA for 48 h, and stained for RAD18. Where indicated, cells were IR-irradiated 2 h before fixation. The scale bar corresponds to 10 μm. (**G**) Quantification of (F), RAD18 foci count is shown (mean + SD, *n* = 3, two-tailed *t*-test). (**H**) U2OS cells were treated with control or RNF8 siRNA 48 h and transfected with FLAG-RNF168 plasmid 19 h before IR-irradiation. Cells were fixed after 2 h and stained for FLAG and RAD18. The scale bar corresponds to 10 μm. (**I**) Quantification of (H), RAD18 foci count in the FLAG positive and negative cells is shown (mean + SD, *n* = 3, two-tailed *t*-test). (**J**) Co-immunoprecipitation of Ub-H2A form HEK293 cells transfected with EGFP–RAD18–WT, EGFP–RAD18–ΔUBZ or EGFP empty vector control. Where indicated, plasmids were co-transfected with FLAG–RNF168 plasmid. Cells were lysed 24 h after transfection, treated with bensonase and incubated with GFP trap. Co-precipitated H2A was analyzed using immunoblotting. A representative experiment from two repeats is shown. (**K**) Schematic representation of variants in RAD18 protein sequence. Bottom, a heatmap showing phenotype of RAD18 variants in four different assays (from Supplementary Figure S3). The RAD18-ΔUBZ is included in the scheme as a negative control. The color legend is normalized to range from 0 (empty EGFP vector) to 1 (wild-type RAD18).

To investigate RAD18 function in DSB repair we depleted endogenous RAD18 in U2OS cells expressing the traffic light reporter that allows monitoring of DNA repair pathway choice by flow cytometry (Supplementary Figure S1A) (35). Consistent with previous reports we observed that depletion of RAD18 reduced efficiency of DNA repair by homologous recombination to ∼60% (Figure 1A, Supplementary Figure S1B) (19,37). Interestingly, the impaired HR was compensated by elevated NHEJ suggesting that RAD18 controls equilibrium between NHEJ and HR without dramatically affecting the total repair efficiency (Figure 1A, Supplementary Figure S1C). Next, we generated U2OS–RAD18–KO cells and confirmed that loss of RAD18 impaired survival upon exposure to UVC and IR, which is consistent with expected defect in TLS and DSB repair (Supplementary Figure S1D, E). Since RAD18 has been suggested to modulate DNA damage signaling, we tested activation of ATM in parental U2OS and U2OS–RAD18–KO cells exposed to ionizing radiation (38). Importantly, we detected no major changes suggesting that loss of RAD18 does not interfere with recognition of DSBs (Supplementary Figure S1F). Quantitative high-content microscopy revealed that U2OS–RAD18–KO cells have impaired RAD51 recruitment to IR-induced foci as well as decreased resection of DSBs ends as detected by native BrdU staining, confirming the defect in HR (Figure 1B, C). Inversely, we observed that recruitment of 53BP1 to DNA damage foci was increased in U2OS–RAD18–KO cells compared to parental U2OS cells, revealing that RAD18 limits binding of 53BP1 to chromatin at endogenous levels (Figure 1D). Importantly, increased recruitment of 53BP1 in U2OS–RAD18–KO cells was rescued by expression of the wild-type RAD18 confirming the specificity of the observed phenotype (Figure 1E, Supplementary Figure S1G, H). In contrast, RAD18-ΔUBZ mutant did not rescue 53BP1 recruitment confirming that the UBZ domain is involved in counteracting 53BP1 function at chromatin (Figure 1E, Supplementary Figure S1G, H). Increased recruitment of 53BP1 to DSBs was observed also upon RNAi-mediated depletion of endogenous RAD18 in U2OS, MCF7 and RPE cells indicating that the impact of RAD18 on 53BP1 function is not restricted to one experimental model (Supplementary Figure S2A, B). Overall, our observations with depletion or deletion of RAD18 fully confirmed previous reports that demonstrated the ability of over-expressed RAD18 to counteract formation of 53BP1 foci (22). In contrast, we did not find any positive effect of RAD18 on promoting 53BP1 retention at chromatin (24). To exclude the possibility that our observations were biased by analyzing heterogeneous cellular populations, we quantified 53BP1 foci in individual cell cycle phases. Although the increase in 53BP1 foci count was more pronounced in the S and G2/M phases in RAD18–KO cells, the same trend was followed even in G1 confirming that effect of RAD18 to 53BP1 accumulation is overall negative. (Supplementary Figure S2C).

RAD18 localization to DNA damage foci was shown to be dependent on chromatin ubiquitination (19,39). Accordingly, we found that depletion of RNF8 or RNF168 ubiquitin ligases impaired formation of RAD18 and 53BP1 foci in both non-treated cells and cells exposed to IR (Figure 1F, G, Supplementary Figure S2D). *In vitro* binding assays revealed that RAD18 binds polyubiquitin chains as well as monoubiquitinated histone H2A (23,39). Since cooperation of RNF8 and RNF168 results in both polyubiquitinated and monoubiquitinated H2A, the direct evidence for interaction of RAD18 with monoubiquitinated H2A in the cellular context was missing. To eliminate a possible effect of RNF8-catalysed polyubiquitination, we overexpressed RNF168 in RNF8-depleted cells (40,41). In this experimental setup, we observed that RNF168 activity alone was sufficient for RAD18 as well as 53BP1 recruitment (Figure 1H, I, Supplementary Figure S2E). Additionally, we found that the wild-type RAD18 but not RAD18-ΔUBZ mutant efficiently pulled-down monoubiquitinated H2A (Figure 1J). Based on this combined data, we propose that RAD18 predominantly binds to the H2A monoubiquitinated by RNF168.

### RAD18 mutations in cancer patients show defects in recruitment to DSBs

RAD18 can potentially contribute to genome instability and tumorigenesis by several means. On one hand, enhanced TLS and ssDNA gap filling that depends on activity of RAD18 increase the mutation burden due to low fidelity of translesion DNA polymerases (17,26,42). On the other hand, loss of RAD18 impairs the error-free DNA repair by HR and *RAD18^−/−^* tumors show similar mutational signature as the BRCA1-deficient tumors (18). As germline mutations in genes involved in HR repair are relatively common in cancer patients, we decided to exploit data for *RAD18* germline variants identified in cancer patients by the CZECANCA consortium (Supplementary Table S1 and S2) (36). Overall, we identified six protein-truncating germline variants in 13 (0.07%) of the 17 654 cancer patients analyzed, indicating that germline, protein-truncating *RAD18* variants are rare, preventing assessment of their potential impact on the cancer risk in our dataset. In addition to the truncating variants, we identified another 52 rare missense variants of which we subjected nine variants localized in the conserved functional domains of RAD18 to further testing by transfecting the wild-type or mutant EGFP–RAD18 to U2OS–RAD18–KO cells (Figure 1K). The most frequent were the C-terminal missense variants I470M, R476H and R477H found in 10, 53 and 5 analyzed cancer patients, respectively. Out of these, we noticed that R476H and R477H affected the NLS and impaired the nuclear localization of RAD18 (Figure 1K, Supplementary Figure S3A). Due to the defect in nuclear localization, R476H and R477H variants also showed impaired ability to ubiquitinate PCNA after exposure to UV and to counteract formation of 53BP1 foci after IR (Figure 1K, Supplementary Figure S3B, C). In addition, we identified three variants localized in the UBZ domain (residues 201–228), namely H219Y, R226C, E227K and we compared them to the wild-type RAD18 and to the RAD18-ΔUBZ mutant. Interestingly, H219Y and R226C mutants showed impaired recruitment to IR-induced foci and they failed to counteract the 53BP1 localization (Figure 1K  Supplementary Figure S3C, D). In addition, we found that H219Y and R226C showed reduced binding to synthetic K63 ubiquitin chains as well as to ubiquitinated H2A *in vitro* (Supplementary Figure S3E, F). This observation confirms functional importance of H219 that is responsible for coordination of Zn^2+^ ion and of R226 that is located in the UBZ domain (43). In contrast, the ability of the E227K mutant to suppress 53BP1 foci formation was unaffected (Supplementary Figure S3C). We conclude that H219 and R226 residues of RAD18 are essential for its ability to bind ubiquitin and its function at DSBs.

### RAD18 localizes in the center of DSB-induced nuclear foci

**Figure 2.**
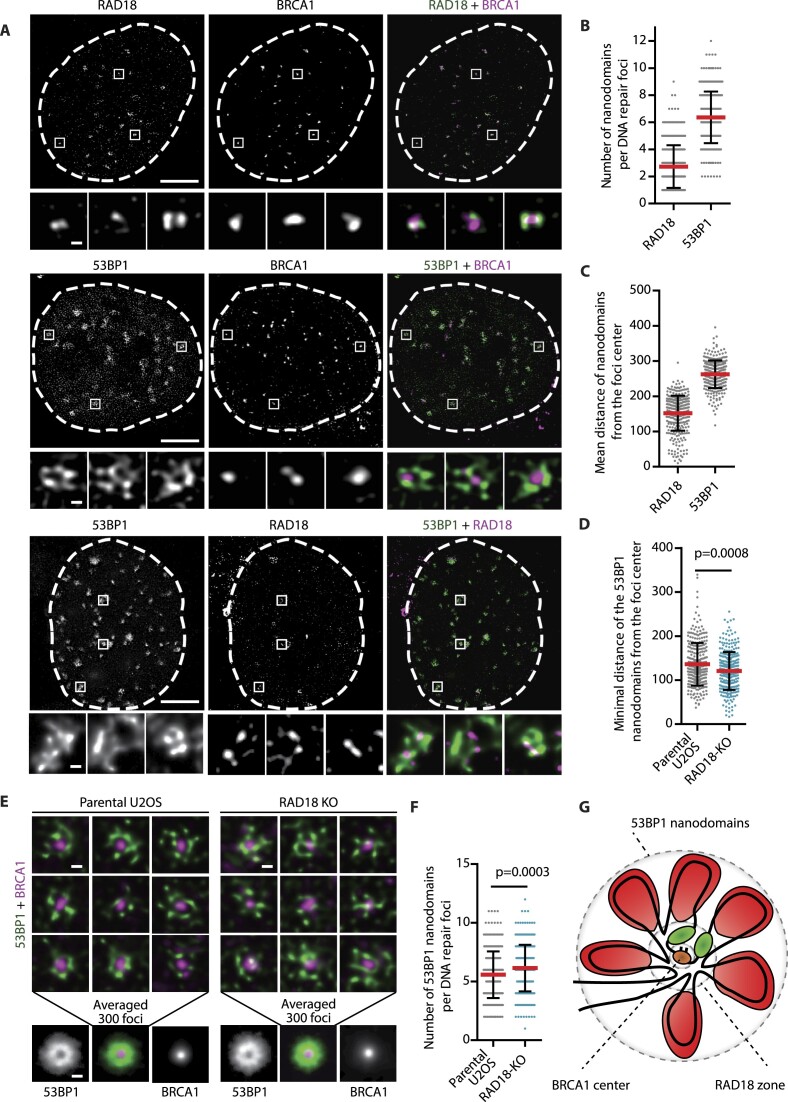
RAD18 excludes 53BP1 to the periphery of IR-induced foci. (**A**) STED images of U2OS cells fixed 3 h after IR-irradiation with 2 Gy and stained with indicated antibodies (scale bars 10 μm and 200 nm). (**B**) Quantification of A, number of RAD18 and 53BP1 nanodomains in individual repair foci. (**C**) Quantification of (A), mean distance of RAD18 and 53BP1 nanodomains from the foci center. For (B) and (C), the mean ± SD is shown, *n* ≥ 311 foci from 26 cells from two independent experiments. (**D**) Quantification of (E), minimal distance of RAD18 and 53BP1 nanodomains from the foci center. The mean ± SD is shown, Mann–Whitney test, *n* ≥ 300 foci form 26 cells from two independent experiments. (**E**) STED images of DNA repair foci in parental U2OS and RAD18–KO cells fixed 3 h after IR-irradiation with 2 Gy and stained with BRCA1 and 53BP1 antibodies. Bottom, signal average is shown of 300 foci (scale bar 200 nm). (**F**) Quantification of E, 53BP1 nanodomains count in individual repair foci. The mean ± SD is shown, Mann–Whitney test, *n* ≥ 300 foci form 26 cells from two independent experiments. (**G**) Model showing arrangement of BRCA1 (brown), RAD18 (green) and 53BP1 (red) nanodomains at chromatin loops formed around DSB.

Although both being dependent on the same histone modification, we noticed that RAD18 and 53BP1 foci induced by RNF168 overexpression showed distinct patterns. Co-staining of both proteins revealed that RAD18 occupies the center whereas 53BP1 localizes at the edges of the foci (Supplementary Figure S4A, B). To investigate whether the same organization is adopted also in the IR-induced foci, we used dual-channel STED microscopy and imaged U2OS cells upon exposure to IR. Consistent with previous reports, we observed that BRCA1 localized close to the centers of the foci, whereas 53BP1 formed several nanodomains at the periphery of the foci (Figure 2A–C) (44). Moreover, we noted that RAD18 localized within 2–3 nanodomains positioned close to the BRCA1 signal and was surrounded with multiple 53BP1 nanodomains (Figure 2A–C). Interestingly, we found that the minimal distance between 53BP1 nanodomains and the BRCA1-positive center was significantly reduced in U2OS–RAD18–KO cells (Figure 2D, E). In addition, U2OS–RAD18–KO showed slightly elevated mean number of 53BP1 nanodomains per foci (Figure 2F). Interestingly, changes in the foci topology observed in RAD18–KO cells were rescued by expression of EGFP–RAD18 WT but not by the R226C mutant that shows impaired localization to foci (Supplementary Figure S4C). These data indicate that RAD18 localizes between the peripheral 53BP1 and the central BRCA1 compartments of the break (Figure 2G). By spatially limiting 53BP1 activity, RAD18 would control the length of DNA resection.

### Auto-ubiquitination of RAD18 interferes with its localization to DSBs

**Figure 3.**
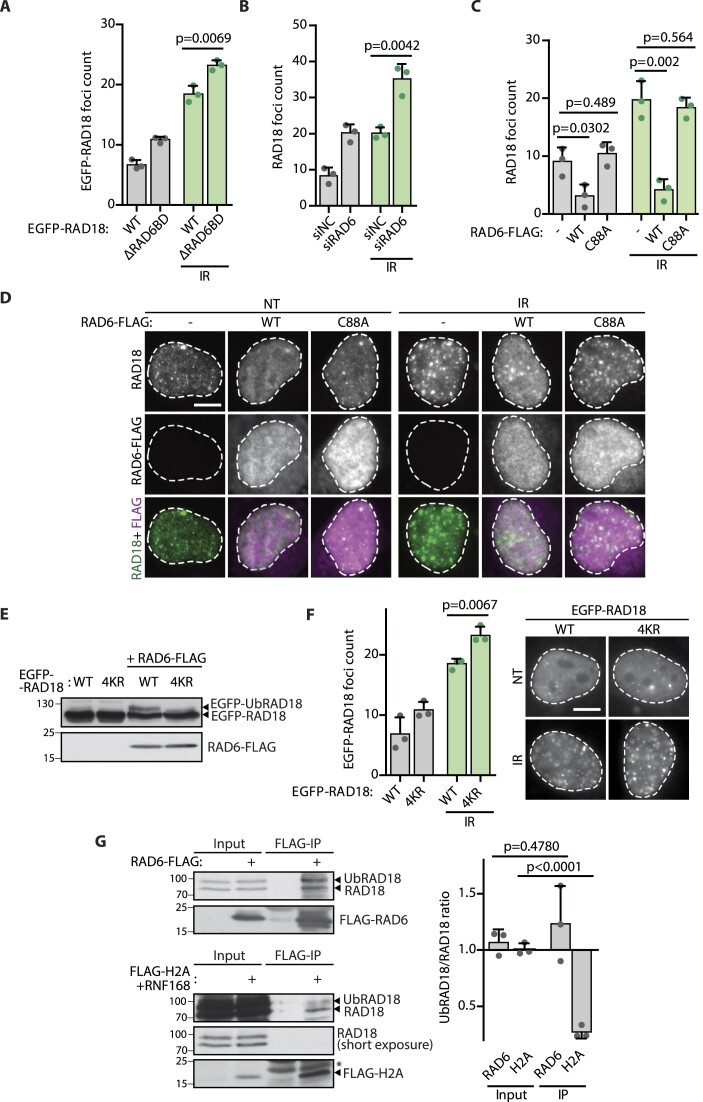
Auto-ubiquitination of RAD18 interferes with its localization to DSBs. (**A**) Quantification of EGFP–RAD18 foci in U2OS RAD18–KO cells transfected with EGFP–RAD18–WT or EGFP–RAD18–ΔRAD6BD. Cells were IR-irradiated 2 h before fixation (mean + SD, *n* = 3, two-tailed *t*-test). (**B**) Quantification of RAD18 foci in U2OS cells treated with control or RAD6 siRNA for 48 h. Where indicated, cells were IR-irradiated 2 h before fixation (mean + SD, *n* = 3, two-tailed *t*-test). (**C**) Quantification of (D), RAD18 foci in U2OS cells transfected or not with RAD6–WT–FLAG or catalytically inactive RAD6–C88A–FLAG mutant. Where indicated, cells were IR-irradiated 2 h prior fixation (mean + SD, *n* = 3, two-tailed *t*-test). (**D**) U2OS cells were transfected with RAD6–WT–FLAG or RAD6–C88A–FLAG. Where indicated, cells were IR-irradiated 2 h prior fixation. The scale bar corresponds to 10 μm. (**E**) U2OS cells were transfected with EGFP–RAD18–WT or EGFP–RAD18–4KR mutant. Where indicated, cells were co-transfected with RAD6–FLAG plasmid. RAD18 and UbRAD18 levels were analyzed using immunoblotting. A representative experiment form two independent repeats is shown. (**F**) Quantification of EGFP–RAD18 foci in U2OS RAD18–KO cells transfected with EGFP–RAD18–WT or EGFP–RAD18–4KR. Where indicated, cells were IR-irradiated 2 h before fixation (mean + SD, *n* = 3, two-tailed *t*-test). The scale bar in the representative image corresponds to 10 μm. (**G**) HEK293 cells were transfected with a plasmid coding for RAD6–FLAG (top) or co-transfected with FLAG–H2A and RNF168 plasmids (bottom). Cells were lysed 24 h after transfection, treated with bensonase and immunoprecipitated with FLAG antibody. Co-precipitated RAD18 and UbRAD18 were analyzed using immunoblotting. Asterisk indicate a non-specific signal of antibody light chains. Right, immunoblot quantification of UbRAD18/RAD18 ratio (mean ± SD, *n* = 3, two-tailed *t*-test).

Negative regulation of 53BP1 was shown to be independent of the catalytic activity of RAD18 (21,22). Nevertheless, it was proposed that RAD18–RAD6 might ubiquitinate 53BP1 and other proteins at DSBs including H2A (24,45–48). First, we asked if RAD18 could act by regulating the stability of 53BP1 but we did not observe any changes in 53BP1 levels in parental, U2OS–RAD18–KO and RPE–RAD18–KO cells (Supplementary Figure S5A). To test whether RAD18 is recruited to DSBs in complex with its essential cofactor RAD6, we evaluated localization of EGFP-tagged RAD6 at DSBs in the parental and U2OS–RAD18–KO cells. Surprisingly, we did not observe enrichment of RAD6-EGFP in IR-induced foci (Supplementary Figure S5B). In addition, we found that EGFP–RAD18–ΔR6BD mutant that is deficient in binding of RAD6 localized to the foci upon exposure to IR, confirming that interaction with RAD6 is not required for RAD18 recruitment to DSBs (Figure 3A). In fact, we observed a slightly increased number of EGFP–RAD18–ΔR6BD foci compared to the wild-type EGFP–RAD18 (Figure 3A). Similarly, siRNA depletion of RAD6 enhanced recruitment of RAD18 to IR-induced foci in three different cell lines, suggesting that the pool of RAD18 that accumulates at DSBs is distinct form the pool of RAD18 restricted to the RAD18_2_RAD6 complex (Figure 3B, Supplementary Figure S5C, D). As consequence of enhanced RAD18 recruitment, we observed decreased 53BP1 accumulation to IR-induced foci in RAD6-depeted cells (Supplementary Figure S5E). We then asked whether the enzymatic activity of RAD6 is required to prevent RAD18 from accumulating at DSBs. We tested this possibility by overexpressing the wild-type RAD6–WT–FLAG or enzymatically deficient RAD6–C88A–FLAG mutant in cells. Interestingly, whereas RAD6–WT–FLAG strongly suppressed RAD18 foci formation, the RAD6–C88A–FLAG mutant did not affect the ability of RAD18 to form IR-induced foci (Figure 3C, D). This phenotype cannot be explained by reduced RAD18 stability or its translocation to the cytoplasm, because RAD6 overexpression had no significant effect on RAD18 nuclear intensity and nuclear/cytoplasmic signal ratio (Supplementary Figure S5F). Enzymatic activity of RAD18 leads to its auto-ubiquitination that can be detected as slower migrating band on SDS-PAGE, however the modified residue (s) have yet not been identified (Supplementary Figure S5G) (49–51). Based on alignment of the RAD18 AlphaFold model with available crystal structures of isolated RAD18 domains, RAD6 and ubiquitin, we predicted lysine residues 186, 197, 201 and 218 to be accessible for ubiquitin conjugation from the active site cysteine of RAD6 (Supplementary Figure S5H). We generated a RAD18-4KR mutant where four of the potentially ubiquitinated residues were mutated to arginines and observed that its ubiquitination was strongly reduced (Figure 3E). Interestingly, RAD18-4KR mutant formed significantly more foci than the wild-type RAD18 supporting the possibility that RAD18 auto-ubiquitination interferes with the ability to bind to the damaged chromatin (Figure 3F). In agreement with this observation, we found that histone H2A preferentially interacted with the faster migrating hence non-ubiquitinated form of RAD18 (Figure 3G) (49). This behavior was in clear contrast to RAD6 that interacted with both RAD18 species. As the UBZ domain of RAD18 is needed for its interaction with chromatin (19,39), it is plausible that binding of UBZ domain to the ubiquitin conjugated to RAD18 might reduce the availability of the domain for binding the ubiquitinated histones.

### SLF1 promotes RAD18 binding to the post-replicative chromatin

**Figure 4.**
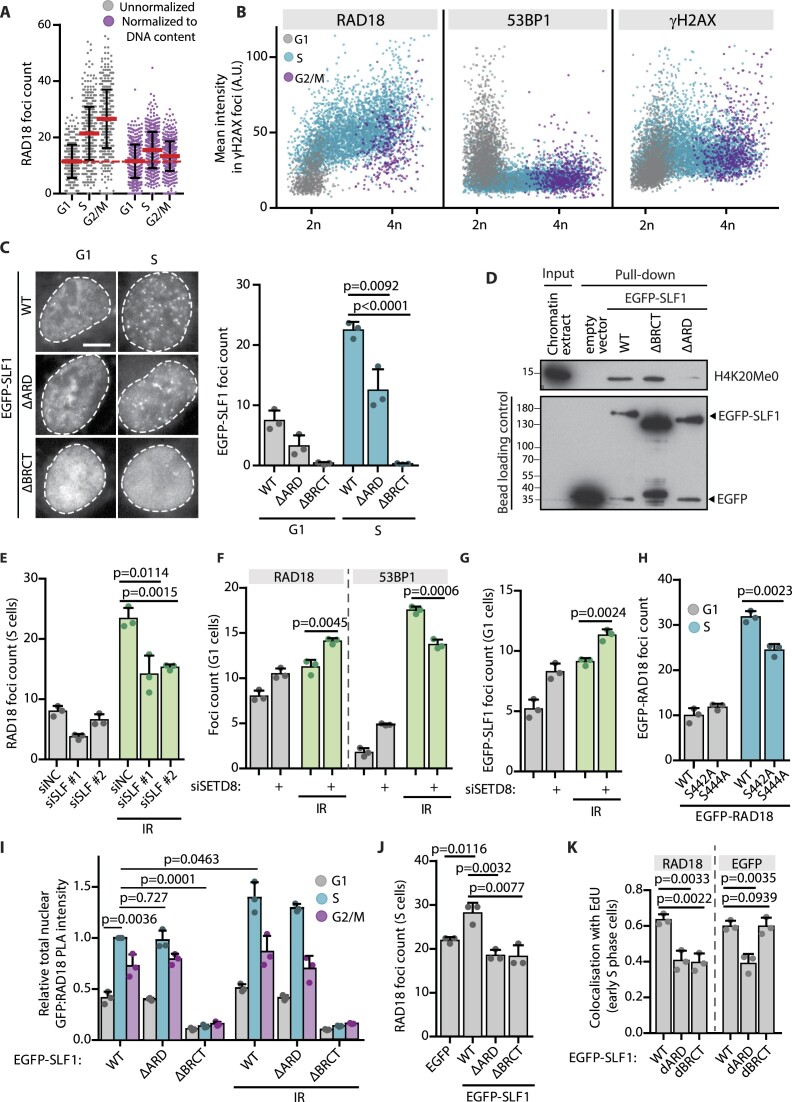
SLF1 promotes RAD18 binding to the post-replicative chromatin. (**A**) Quantification of RAD18 foci in U2OS fixed 2 h upon IR-irradiation. Cell cycle phases were distinguished based on the total DAPI and EdU nuclear intensities. The foci count was normalized to the total DAPI nuclear intensity of 2*n* cells (mean ± SD, *n* = 514). (**B**) Quantification of mean RAD18/53BP1/γH2AX intensity in γH2AX foci plotted against the total nuclear DAPI intensity.) RPE-iCut cells were treated overnight with Shield-1 and doxycycline to induce expression of Cas9 endonuclease that was targeted to DNA upon transfection of mixture of 12 single cutter sgRNAs. Cells were fixed 6 h post transfection. Color legend shows cell cycle phases distinguished by gating on the total EdU and DAPI nuclear intensities (mean ± SD, *n* ≥ 4700). A representative experiment form two independent repeats is shown. (**C**) U2OS cells transfected with wild-type EGFP-SLF1 or ΔBRCT and ΔARD variants were fixed 2 h after IR-irradiation. The scale bar in the representative image corresponds to 10 μm. Right, quantification shows EGFP-SLF1 foci count in G1 and S cell cycle phases distinguished by gating on the total EdU and DAPI nuclear intensities (mean + SD, *n* = 3, two-tailed *t*-test). (**D**) HEK293 cells were transfected with the wild-type EGFP-SLF1 or ΔBRCT and ΔARD variants, or EGFP empty vector control. Cells were lysed 24 h after transfection, treated with bensonase, incubated with GFP trap and extensively washed. Immunoprecipitated EGFP-SLF1 was then incubated with acid histone extracts. Pulled-down H4K20me0 was analyzed using immunoblotting. A representative experiment form two independent repeats is shown. (**E**) Quantification of RAD18 foci count in EdU positive U2OS cells treated with control or two different SLF1 siRNAs for 48 h. Where indicated, cells were IR-irradiated 2 h before fixation (mean + SD, *n* = 3, two-tailed *t*-test). (**F**) Quantification of RAD18 and 53BP1 foci count in U2OS cells treated with control or SETD8 siRNA for 24 h. Where indicated, cells were IR-irradiated 2 h before fixation. G1 cells were distinguished by gating on the total EdU and DAPI nuclear intensities (mean + SD, *n* = 3, two-tailed *t*-test). (**G**) Quantification of EGFP-SLF1 foci in G1 cells transfected with EGFP-SLF1 and treated as in F and G (mean + SD, *n* = 3, two-tailed *t*-test). (**H**) Quantification of EGFP–RAD18 foci count in U2OS–RAD18–KO cells transfected with EGFP–RAD18–WT or EGFP–RAD18–S442/444A and fixed 2 h after IR-irradiation. G1 and S cells were distinguished by gating on the total EdU and DAPI nuclear intensities (mean + SD, *n* = 3, two-tailed *t*-test). (**I**) PLA quantification of RAD18–GFP–SLF1 interaction. U2OS cells were transfected with SLF1 variants and IR-irradiated as indicated 2 h prior fixation. Cells were then probed for PLA with RAD18 and GFP antibodies. Relative total nuclear PLA intensity is shown that is normalized to intensity of SLF1–WT in S cells (mean + SD, *n* = 3, single-sample two-tailed *t*-test). (**J**) Quantification of RAD18 foci in EdU positive U2OS cells transfected with wild-type EGFP-SLF1 or ΔBRCT and ΔARD variants and IR-irradiated 2 h before fixation (mean + SD, *n* = 3, two-tailed *t*-test). (**K**) Colocalization of RAD18 and EGFP with EdU in U2OS cells transfected with EGFP-SLF1 variants. Colocalization in early S cells was evaluated by Pearson's correlation coefficient. At least 26 cells per condition were analyzed in three independent experiments (mean + SD, two-tailed *t*-test).

To investigate differences in RAD18 function in DSB repair throughout the cell cycle, we imaged asynchronically growing cells, and determined their cell cycle phases and number of RAD18 foci formed upon exposure to IR (Figure 4A). Since irradiation-induced DNA damage load is linearly proportional to the DNA content, we normalized RAD18 foci count to the total DAPI intensity (19,52). We found that the highest number of RAD18 foci was present in the S phase cells, which is consistent with its function in HR (Figure 4A). However, we cannot exclude possible contribution of replication-dependent recruitment of RAD18, as IR causes various types of DNA lesions potentially inducing collapse of replication forks. To evaluate RAD18 accumulation specifically at DSBs we induced Cas9 expression in RPE-iCut cells and transfected them with a mixture of 12 single-cutter sgRNAs (31). Consistently with previous findings, RAD18 accumulated at γH2AX foci most efficiently in S/G2 cells (Figure 4B). In contrast, 53BP1 foci accumulation peaked in G1 phase and γH2AX was phosphorylated independently to the cell cycle phase (Figure 4B, Supplementary Figure S6A). Cell cycle-specific recruitment of DNA repair proteins to chromatin can be directed by a variable histone H4 methylation status. Whereas H4K20Me2 is recognized by the tandem tudor domain of 53BP1 (9,53), the newly incorporated H4K20Me0 into DNA during replication is recognized by the ARD domains of TONSL and BARD1 (10,11). Additionally, a highly conserved ARD domain has been identified in the SMC5-SMC6 complex localization factor protein 1 (SLF1, also known as BRCTx, ANKRD32, or BRCTD1) and was shown to be capable of H4K20me0 binding *in vitro* (10). SLF1 forms complex with SLF2-SMC5/6 and it was shown to interact with RAD18 phosphorylated at S442 and S444 through its tandem BRCT domain (29,54,55). In contrast to the RAD18–RAD6 complex, binding of the RAD18–SLF1/2–SMC5/6 complex at the chromatin was reported to be ubiquitin-dependent (29). We hypothesized, that the UBZ and ARD domains of the RAD18-SLF1 complex might serve as a bivalent reader of H2AK15Ub and H4K20Me0 in the manner similar to the ARD and BRCT domains of BARD1. By imaging of the cells transfected with EGFP-SLF1, we established that SLF1 is recruited to IR-induced nuclear foci preferentially during the S phase which correlates with elevated H4K20Me0 during DNA replication (Figure 4C, Supplementary Figure S6B). This localization was dependent on ARD as well as BRCT domains of SLF1 (Figure 4C). Moreover, deletion of ARD domain impaired co-immunoprecipitation of histone H4K20Me0 with SLF1 supporting the previous conclusions from *in vitro* assays (Figure 4D). Further, we found that depletion of SLF1 reduced formation of RAD18 foci in U2OS, MCF7 and RPE cells during the S phase and inversely, it increased formation of 53BP1 foci (Figure 4E, Supplementary Figure S6C, S6D, S6E). Moreover, we found that SLF1-depleted U2OS cells showed increased sensitivity to IR, probably reflecting dysregulated DNA repair (Supplementary Figure 6F) (29). To increase levels of H4K20Me0, we depleted the methyltransferase SETD8 (also known as KMT5A) that initiates the methylation of H4K20 after replication (56). Since exhaustive SETD8 depletion interferes with replication and introduces DNA damage (Supplementary Figure S6G) (57), we analyzed cells 24 h after siRNA transfection. This treatment was sufficient to elevate levels of H4K20Me0 in the pre-replicative chromatin of the G1 cells (Supplementary Figure S6H). Importantly, SETD8 depletion increased formation of EGFP-SLF1 and RAD18 foci and, inversely, it reduced recruitment of 53BP1 (Figure 4F, G, Supplementary Figure S6I). Next, we confirmed that substitution of RAD18 serine residues 442/444 to alanines disrupted the interaction with SLF1 (Supplementary Figure S7A). In addition, EGFP–RAD18–S442/444A mutant showed reduced formation of RAD18 foci specifically in the S phase, further supporting the model in which SLF1 promotes RAD18 recruitment to the post-replicative chromatin (Figure 4H). The proximity ligation assay showed that the SLF1-ΔBRCT mutant was unable to interact with RAD18 and it also failed to promote RAD18 recruitment to IR-induced foci (Figure 4I, J). Further, we found that SLF1-ΔARD mutant showed reduced localization at the post-replicative chromatin labeled by incorporation of EdU (Figure 4K, Supplementary Figure S7B). Finally, we observed that SLF1-ΔARD mutant failed to promote RAD18 foci formation confirming that not only SLF1–RAD18 interaction mediated by BRCT domain, but also the ability of the ARD domain to bind H4K20Me0 are necessary for full SLF1–RAD18 recruitment to DSBs (Figure 4I, J). Both SLF1 and RAD18 contribute differently in their cooperative binding. Whereas the ARD domain endows the SLF–RAD18 complex with specificity for the post-replicative chromatin, the DSBs recruitment is dependent on the RAD18 UBZ domain. As a consequence, RAD18 is inefficiently recruited to the post-replicative chromatin in SLF1-depleted cells (Supplementary Figure S6C) and SLF1 accumulation at DSBs induced by IR or CRISPR/Cas9 is fully abolished in RAD18–KO cells (Supplementary Figure S7C, D).

Finally, we investigated the changes in colocalization of RAD18 with other DNA repair proteins throughout the cell cycle. As expected, we observed high level of colocalisation between RAD18 and γH2AX or conjugated ubiquitin stained with FK2 antibody. This overlap was apparent in all cell cycle phases, confirming that formation of RAD18 foci depends on activation of ATM and protein ubiquitination (Supplementary Figure S7E, F). In contrast, colocalisation of RAD18 with 53BP1 and BRCA1 markers showed inversed trends during the cell cycle (Supplementary Figure S7F). Whereas colocalisation of RAD18 and 53BP1 was high in G1 cells, it dropped to 40% in S phase cells (Supplementary Figure S7F). In contrast, there were almost no double positive foci for RAD18 and BRCA1 in G1 cells but the colocalisation increased to 60% during S phase (Supplementary Figure S7F). In G2 cells, the fraction of RAD18/BRCA1 positive foci decreased, probably reflecting the increased level of histone methylation compared to the S phase. In agreement with our previous observations, depletion of SLF1 and RAD6 showed inversed effects on the formation of RAD18 foci throughout the cell cycle (Supplementary Figure S7F). Interestingly, we noted that SLF1 and RAD6 depletion had a global effect on RAD18 foci, without dramatically changing their colocalization frequency with BRCA1, 53BP1 or γH2AX (Supplementary Figure S7F).

### RAD18 promotes recruitment of SMC5 to DSBs

**Figure 5.**
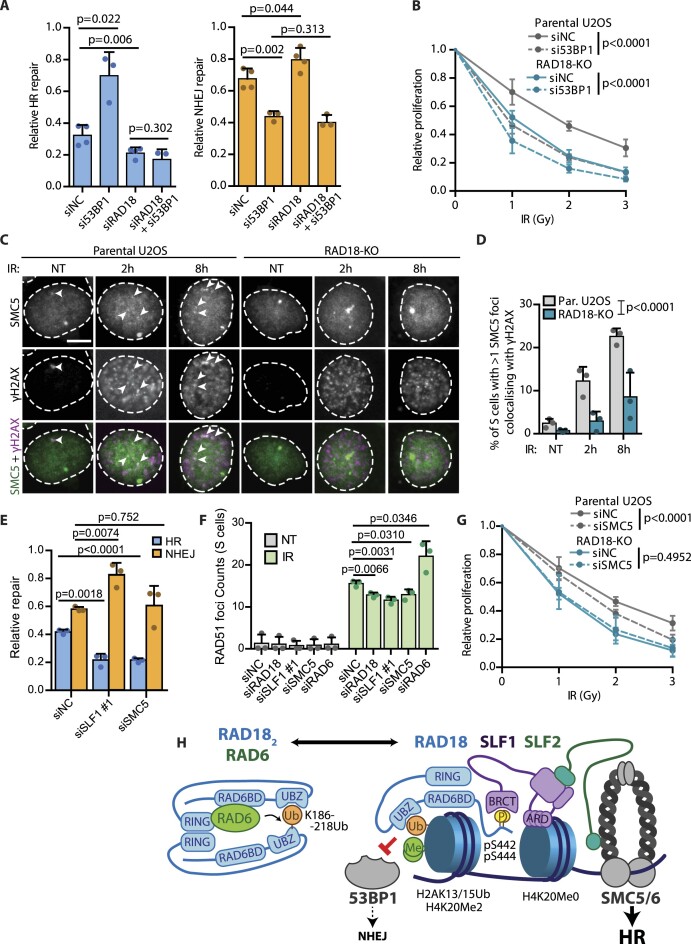
RAD18 promotes recruitment of SMC5 to DNA lesions. (**A**) Relative HR (right) and NHEJ (left) repair in traffic light reporter U2OS cells treated with indicated siRNAs. The repair efficiency was normalized to the total repair efficiency in control siRNA treated cells (mean + SD, *n* ≥ 3, two-tailed *t*-test). (**B**) Relative proliferation of parental U2OS and RAD18–KO cells treated with control or 53BP1 siRNA was evaluated using resazurin viability assay 7 days after IR-irradiation with indicated doses (mean ± SD, *n* ≥ 3, two-way ANOVA). (**C**) Colocalization of SMC5 with γH2AX foci in parental U2OS and RAD18–KO. Cells were incubated with EdU prior IR-irradiation, pre-extracted and fixed at indicated time points. Incorporated EdU was Click-iT-labelled and cells were stained for SMC5 and γH2AX. EdU positive cells are shown (arrows indicate SMC5 foci colocalizing with γH2AX foci, scale bar 10 μm). Right, quantification of cell fraction with >1 SMC5 foci colocalizing with γH2AX foci. EdU positive cells were analyzed (mean + SD, *n* = 3, two-tailed *t*-test). (**D**) Quantification of the mean nuclear SMC5 intensity in parental U2OS and RAD18–KO cells. Where indicated, cells were pre-extracted prior fixation (mean ± SD, *n* = 300, Mann–Whitney test). (**E**) Relative HR and NHEJ repair in traffic light reporter U2OS cells treated with indicated siRNAs. The repair efficiency was normalized to the total repair efficiency in control siRNA treated cells (mean + SD, *n* ≥ 3, two-tailed *t*-test). (**F**) Quantification of RAD51 foci in U2OS cells treated with indicated siRNAs for 48 h. Cells were incubated with EdU prior IR-irradiation, pre-extracted and fixed after 6 h. Incorporated EdU was Click-iT-labelled and cells were stained for RAD51. EdU positive cells were analyzed (mean + SD, *n* = 3, two-tailed *t*-test). (**G**) Relative proliferation of parental U2OS and RAD18–KO cells treated with control or SMC5 siRNA was evaluated using resazurin viability assay 7 days after IR-irradiation with indicated doses (mean ± SD, *n* ≥ 3, two-way ANOVA). (**H**) Model describing RAD18 function at DSBs. RAD18 is recruited to the post-replicative chromatin by bimodal recognition of H2AK15Ub and H4K20me0 with its UBZ domain and with ARD domain of its interacting partner SLF1, respectively. RAD18 then inhibits NHEJ by limiting 53BP1 activity and promotes HR by recruiting the SLF2/SMC5/6 complex. RAD18 accumulation at DSBs is inhibited by RAD6-mediated auto-ubiquitination.

53BP1 limits HR repair by recruiting Rap1-interacting factor 1 homolog (RIF1) that interacts with the shieldin complex containing CST-Polα–primase and actively counteracts DNA end resection (58–60). The unrestricted activity of the 53BP1–shieldin complex could explain impaired HR repair in RAD18 deficient cells and we tested this hypothesis using traffic light reporter assay. Surprisingly, co-depletion of 53BP1 failed to rescue HR efficiency in RAD18 depleted cells (Figure 5A). Although we observed a rescue in HR/NHEJ ratio, this was caused by proportionally decreased efficiency of both pathways (Supplementary Figure S8A). In agreement with that, depletion of 53BP1 further sensitized RAD18 deficient cells to IR (Figure 5B). By limiting 53BP1 activity, RAD18 seems to suppress NHEJ repair without affecting HR efficiency. Indeed, co-depletion of RAD18 and 53BP1 failed to increase NHEJ efficiency suggesting an epistatic behavior of RAD18 and 53BP1 in NHEJ regulation (Figure 5A). These observations implicate that RAD18 promotes HR independently on 53BP1. We hypothesized that this activity might be mediated by the Structural maintenance of chromosomes SMC5/6 complex that was shown to associate with RAD18-SLF1-SLF2 at DNA interstrand crosslinks (29). SMC5/6 is an evolutionary conserved protein complex implicated in DNA repair by homologous recombination, replication fork stabilization and DNA loop extrusion (61–64). In agreement with its multifaceted role, SMC5/6 complex is recruited to DNA by several independent mechanisms (65,66). To investigate the role of RAD18/SLF1/SLF2 axis in SMC5/6 recruitment to the chromatin we first documented enrichment of SMC5 at DSBs in U2OS cells exposed to IR (Figure 5C, Supplementary Figure S8B). The total mean nuclear signal of SMC5 was not affected in pre-extracted U2OS–RAD18–KO cells suggesting that RAD18 is not needed for global chromatin distribution of SMC5 (Supplementary Figure S8C). However, the localization of SMC5 in DNA repair foci was reduced in U2OS–RAD18–KO cells, confirming that RAD18 promotes SMC5 recruitment specifically to DSBs (Figure 5D) (67). Similarly, we observed recruitment of SMC5 to DSBs induced by transfection of RPEi-Cut cells with either mixture of single cutter sgRNAs or sgRNA targeting the 45S rDNA repeats (Supplementary Figure S8D). As expected, we observed impaired recruitment of SMC5 Cas9-induced foci in the absence of RAD18 as well as increased intensity of 53BP1 signal (Supplementary Figure S8D, S8E). Further, we noted that depletion of SLF1 or SMC5 reduced HR efficiency in TLR assay (Figure 5E). Similarly, depletion of RAD18, SLF1 or SMC5 comparably impaired formation of RAD51 foci upon IR exposure further supporting their role in HR (Figure 5F). To document the importance of the SMC5/6 complex in DSB repair, we tested sensitivity of SMC5-depleted cells to IR (29). Importantly, SMC5-depletion did not further sensitize RAD18 deficient cells confirming action of both factors in the same repair axis (Figure 5G). Similarly to distinct recruitment modes of SMC5, RAD18 was not needed for retention of SLF1 at unperturbed chromatin (Supplementary Figure 8F), although being essential for SLF1 accumulation at sites of DNA damage (Supplementary Figure S7C, D). This is in agreement with two modes of recruitment that have been reported for Nse5, a yeast paralog of SLF1 (68). Besides the function in the nucleus, SLF1 has been also reported to localize at the centrosome in human cells (69). Although we observed this localization is dependent on the BRCT domain of SLF1 (Supplementary Figure S8G), it is retained in RAD18–KO cells suggesting a RAD18-independent localization (Supplementary Figure S8H).

In the summary, we show here that RAD18 inhibits DNA repair by NHEJ by suppressing recruitment of 53BP1 to DSBs. In addition, RAD18 contributes to the HR-mediated repair of DSBs by SLF1-dependent recruitment of SMC5/6 complex to the post-replicative chromatin.

## Discussion

Overexpression of RAD18 was shown to limit irradiation-induced 53BP1 foci formation and it was assumed that this was due to a physical competition of ubiquitin-binding domains for H2A-K15Ub (22,23). However, the experimental evidence describing such RAD18 function under physiological conditions was missing. In this study, we documented that endogenous RAD18 inhibits 53BP1 recruitment to DSBs. We show that endogenous RAD18 interacts with H2A ubiquitinated by RNF168. Although RAD18 has been reported to bind K63 ubiquitin chains *in vitro*, we identified that activity of RNF168 is sufficient for recruitment of RAD18 to the IR-induced foci. The possibility that H2A ubiquitinated by RNF168 is the major partner for RAD18 binding is consistent with *in vitro* experiments that revealed high (nanomolar) affinity of the RAD18 UBZ domain to the H2A-K15Ub-containing nucleosomes (∼8-fold higher than affinity of BARD1, 2 fold higher than RNF168, and two orders higher than 53BP1) (7,23). Although both 53BP1 and RAD18 recognize the same ubiquitin mark, their localization in the IR-induced foci structure is different. Using STED microscopy, we show that RAD18 occupies 2–3 chromatin nanodomains close to the resection center. In contrast, 53BP1 occupies 5–6 nanodomains at the foci periphery (44). This spatial organization can likely result from progressive spreading of ubiquitin signaling from the DNA break to the peripheral chromatin. RAD18 readily covers ubiquitinated chromatin at the vicinity of the foci center. Once the local pool of free RAD18 is exhausted, chromatin may become accessible for 53BP1 that binds ubiquitinated nucleosomes with micromolar affinity. The peripheral localization of 53BP1 domains is further augmented by a chromatin remodeler SMARCAD1 acting as reader of BRCA1/BARD1-mediated ubiquitination (70). Localization of RAD18 is similar to the positioning suggested for RNF169, another factor restricting 53BP1 foci accumulation (71). We propose that RAD18 and RNF169 may create a buffering zone between the peripheral 53BP1 and the central BRCA1 compartments of the break. By spatially limiting 53BP1 activity, they would control extent of DNA resection. RAD18 shows remarkably strong binding to H2AK15Ub. To enable simultaneous recruitment of all H2A-K15Ub readers, RAD18 function needs to be tightly regulated. We propose that this regulation is mediated by RAD6, as its depletion substantially enhances RAD18 recruitment to IR-induced foci. This negative control is dependent on RAD6 catalytic activity suggesting it involves RAD18 auto-ubiquitination. Quantitative microscopy did not identify any significant effect of RAD18 modification on its stability and localization. Instead, it seems that RAD18 auto-ubiquitination directly impairs its interaction with H2AK15Ub. The RAD18 UBZ domain is separated from the dimerization RING domain by an unstructured region spanning residues 90–200 enabling its high mobility. It is possible that ubiquitin covalently attached to the UBZ domain is bound by the other UBZ domain of the RAD18 homodimer in *trans* (Figure 5H). In this scenario, both UBZ domains would be inaccessible for binding to ubiquitinated H2A.

Based on the important function of the UBZ domain, we focused on examination of RAD18 variants identified in Czech cancer patients that showed impaired accumulation to IR-induced foci. The defect observed in the C-terminal variants was caused indirectly by disruption of their nuclear localization signal. On the other hand, mutation of the H219 directly impaired UBZ domain function. Although the RAD18 UBZ domain is dispensable for PCNA ubiquitination *in vitro* (51,72), its function becomes essential in the cellular context (22,49). The reason of this discrepancy remains to be solved, however, it is unlikely that the UBZ would be responsible for recruitment of RAD18 to stalled replication forks (73). The split-of-function mutations R226C and E227K further suggests that roles of the UBZ domain in PCNA ubiquitination and DSB repair are independent. The clinical importance of germline *RAD18* variants is currently unknown. The prevalence of truncating or functionally-impaired *RAD18* germline variants was too low in our dataset of the cancer patients and controls to evaluate their association with the risk of individual cancer diagnoses. Nevertheless, we noted increased moderate risks close to statistical significance in patients with endometrial tumors (OR = 3.33; 95% CI 0.94–11.83; *P* = 0.049) and ovarian cancer (OR = 2.67; 95% CI 0.91–7.87; *P* = 0.063). Moreover, there is growing evidence that deregulation of the RAD18 function shapes the mutational landscape of the cancer cells (17,18).

One of the important findings of this study is the observation that RAD18 is specifically recruited to the ubiquitinated chromatin in S/G2 phases. This specificity relies on the intact ARD domain of the RAD18-interacting partner SLF1. Our observations can be likely explained by recognition of the H4K20me0 histone by the highly conserved ARD domain of SLF1 in a similar manner as was described for BARD1 and TONSL (10–12). This hypothesis is supported by the pull-down of H4K20me0 by SLF1 and by its enhanced recruitment upon depletion of the histone methyltransferase SETD8. Interestingly, although they both have distinct specificities for H4K20 methylation status, SLF1 contributes to restriction of 53BP1 from IR-induced foci in the post-replicative chromatin. This suggests a scenario where the newly-incorporated nucleosome carrying H4K20Me0 is recognized by SLF1 and subsequently RAD18 displaces 53BP1 from the adjacent ubiquitinated and parentally-methylated nucleosome (Figure 5H). Such ‘bridge’ model of nucleosome binding has been recently reported for BARD1-BRCA1 enabling direction of the BRCA1 catalytic activity to H4K20me2-carrying nucleosomes (74,75). Simultaneous interaction of BARD1-BRCA1 with two adjacent nucleosomes is facilitated by the flexible intrinsically disordered region (IDR) that connects the ARD domain and the RING domain of BARD1. Similarly, the ARD and BRCT domains of SLF1 are separated by two potentially disordered regions, as predicted by AlphaFold. Besides the similarities described above, there are also differences between BARD1-BRCA1 and SLF1–RAD18 complexes resulting from their different binding affinities. Whereas highly conserved ARD domains of BARD1 and SLF1 provide the complexes with comparable selectivity to H4K20me0, the affinity on the RAD18 UBZ domain towards Ub-H2A is significantly higher compared to the RING domain of BARD1 (7,10,23,76). As consequence, RAD18 can be recruited to DNA lesions in the absence of SLF1 and can partially inhibit 53BP1 in G1 phase. The ARD domain becomes important during the S phase, when it strengthens RAD18-SLF1 association with chromatin, potentially increases its residence time and endows it with specificity to the post-replicative chromatin. On the other hand, BRCA1–BARD1 binding fully relies on cooperative binding of both RING and ARD domains strictly limiting function of this complex to the S phase (10). Another difference between RAD18–SLF1 and BRCA1/BARD1 complexes is represented by their various dependencies on protein phosphorylation. Whereas the interaction between BARD1 and BRCA1 is constitutive, formation of the SLF1–RAD18 complex requires phosphorylation of RAD18 (54,77). Our data show that interaction between SLF1 and RAD18 increases during the S phase and is further boosted by ionizing radiation. Therefore, we speculate that a basal modification of RAD18 might be enhanced by a protein kinase responsive to DNA damage and it will be interesting to address this possibility by future research.

Finally, we show that by limiting 53BP1 access to DSBs, RAD18 inhibits NHEJ without affecting HR repair. This mechanism is different from RAD18 overexpression that was reported to promote Cas9-mediated homology-directed repair by complete inhibition of 53BP1 function (21). Instead, RAD18 likely promotes DSB repair by recruiting positive HR regulators including RAD51C (19). In addition, we propose that endogenous RAD18–SLF1 complex facilitates HR repair by recruiting the SMC5/6 complex (Figure 5H). Accordingly, SMC5 depletion does not further sensitize RAD18–KO cells to IR. The SMC5/6 complex is known to promote sister chromatid recombination during the G2/M, although the precise mechanism is not that clear (64,78–80). Recent data show that Nse5/6, yeast homologs of SLF1/SLF2, inhibit ATPase and loop extruding activities of SMC5/6 (81,82). Instead, Nse5/6 enable high-salt-resistant association of SMC5/6 at chromatin potentially by topological entrapment of DNA (82). This binding mode could promote HR by stabilization of recombination intermediates. Our observation that RAD18 is not needed for loading of SMC5/6 to unperturbed chromatin is in agreement with a recent finding that SMC5/6 is loaded at cohesin-dependent chromosome loop boundaries by specific recognition of positively supercoiled DNA that is generated by transcription (83–85). Taken together, we present a model where the RAD18–SLF1 complex is recruited to the post-replicative DSBs by simultaneous recognition of H2A-K15Ub and H4K20Me0. Subsequently, RAD18 modulates DNA repair by regulating 53BP1 and SMC5/6 accumulation in the proximity of DNA breaks. Combined defects in translesion DNA synthesis, DNA gap filling and DSB repair caused by the loss of RAD18 function may contribute to genome instability in cancer cells.

## Supplementary Material

gkae499_Supplemental_Files

## Data Availability

All data is included in the manuscript and supplementary files. Raw and deconvolved super-resolution images as well as ImageJ and R scripts used for their analysis were uploaded to BioStudies repository (https://www.ebi.ac.uk/biostudies/studies/S-BSST1239) with accession number S-BSST1239. Raw data from high-content microscopy are available from the corresponding author upon request.

## Appendix

Additional members of the CZECANCA consortium who participated in mutation analysis and are not mentioned in author list: Marta Cerna, Klara Horackova, Milena Hovhannisyan, Marketa Janatova, Sandra Jelinkova, Petr Nehasil, Jana Soukupova, Barbora Stastna, and Petra Zemankova (Institute of Medical Biochemistry and Laboratory Diagnostics, First Faculty of Medicine and General University Hospital in Prague, Prague, Czech Republic); Lenka Foretova and Eva Machackova (Department of Cancer Epidemiology and Genetics, Masaryk Memorial Cancer Institute, Brno, Czech Republic); Vera Krutilkova and Spiros Tavandzis (AGEL Laboratories, Novy Jicin, Czech Republic); Leona Cerna, Stepan Chvojka and Monika Koudova (GENNET, Prague, Czech Republic); Ondrej Havranek, Jan Novotny, Kamila Vesela and Michal Vocka (Institute of Biology and Medical Genetics, First Faculty of Medicine and General University Hospital in Prague, Prague, Czech Republic); Lucie Hruskova, Renata Michalovska, Denisa Schwetzova, and Zdenka Vlckova (GHC Genetics, Prague, Czech Republic); Monika Cerna, Marketa Hejnalova, Nikol Jedlickova, Ivan Subrt and Tomas Zavoral (Institute of Medical Genetics, University Hospital Pilsen, Pilsen, Czech Republic); Marcela Kosarova and Gabriela Vacinova (Pronatal, Prague, Czech Republic); Maria Janikova, Romana Kratochvilova, Vaclava Curtisova and Radek Vrtel (Department of Medical Genetics, University Hospital Olomouc, Faculty of Medicine and Dentistry, Palacky University, Olomouc, Czech Republic); Ondrej Scheinost and Petra Duskova (Hospital Ceske Budejovice, Ceske Budejovice, Czech Republic); Viktor Stranecky (Department of Paediatrics and Inherited Metabolic Disorders, First Faculty of Medicine and General University Hospital in Prague, Prague, Czech Republic).
