# Supplementary material for: RAD18 directs DNA double-strand break repair by homologous recombination to post-replicative chromatin
Source: Nucleic Acids Res. 2024 Jun 13;52(13):7687–703. doi: 10.1093/nar/gkae499 (PMC11260465; doi:10.1093/nar/gkae499)
Supplement: gkae499_Supplemental_Files [file gkae499_supplemental_files.zip › Supplementary Figures-RESUBMISSION-corrected2.pdf]

RAD18 directs DNA double-strand break repair by homologous recombination to the post-replicative chromatin

Matous Palek[1, 2], Natalie Palkova[1, 2], consortium CZECANCA[1,3], Petra Kleiblova[3], Zdenek Kleibl[3], Libor Macurek[1]

Supplementary information

Supplementary Figure 1. RAD18 promotes DNA repair upon IR

Supplementary Figure 2. RAD18 excludes 53BP1 form ubiquitinated chromatin

Supplementary Figure 3. Screen of RAD18 cancer variants

Supplementary Figure 4. Role of RAD18 in repair foci topology and antibody validation

Supplementary Figure 5. Regulation of RAD18 by RAD6-mediated autoubiquitination

Supplementary Figure 6. RAD18 is recruited to the post-replicative chromatin

Supplementary Figure 7. RAD18 is recruited to DNA lesions with SLF1

Supplementary Figure 8. RAD18 recruits SMC5 to DSBs

Supplementary Fig. 1

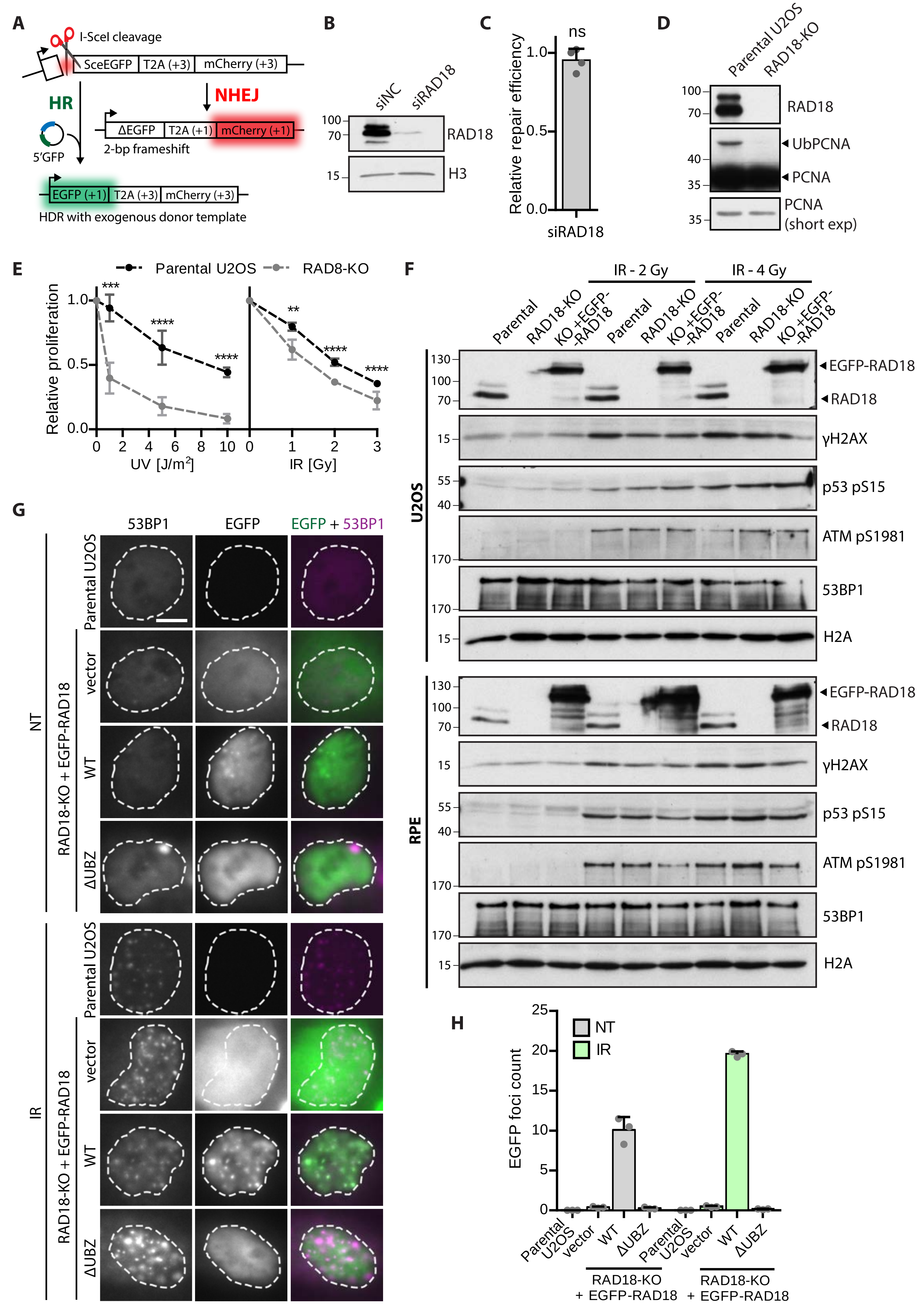

Supplementary Figure 1. RAD18 promotes DNA repair upon IR

(A) Diagram of TLR reporter system. The SceEGFP is a EGFP gene containing an I-SceI site and in-frame termination codon. The SceEGFP is fused to alternative reading frame coding for a ribosome skipping sequence (T2A) and mCherrry in the +3 translation. If the DSB introduced by I-SceI is repaired through the HDR pathway utilizing a plasmid with truncated EGFP donor template, the reporter cells will restore EGFP expression. Alternatively, the break can be repaired by mutagenic NHEJ resulting in +3 frameshift that places T2A in frame and enables mCherry expression.

(B) RAD18 depletion efficiency in reporter U2OS cells was evaluated using immunoblotting.

(C) Total repair efficiency (NHEJ + HR) in RAD18-depleted reporter U2OS cells. Values normalized to control siRNA treated cells are plotted (mean +SD, n = 4, one-sample two-tailed t-test).

(D) Validation of U2OS-RAD18-KO cells by detection of RAD18 and UbPCNA using immunoblotting. Cells were harvested 4 h after UV-irradiation to induce PCNA ubiquitination.

(E) Relative proliferation of Parental U2OS and RAD18-KO cells was evaluated using resazurin viability assay 7 days after irradiation with indicated doses of UV and IR, respectively (mean ±SD is shown, n = 3, two-way ANOVA).

(F) Parental cells, RAD18-KO, and RAD18-KO cells reconstituted with RAD18 WT were harvested 1 h upon irradiation with indicated doses and analyzed by immunoblotting. The upper panel shows U2OS and the lower panel RPE cells.

(G) U2OS parental cells and RAD18-KO cells transfected with wild type EGFP-RAD18-WT, EGFP-RAD18-ΔUBZ, or an EGFP empty vector control were IR-irradiated 24 h post transfection. After 2 h, cells were fixed and stained for 53BP1 (scale bar 10 µm).

(H) Quantification of G, GFP foci count is shown (mean ±SD, n=3, two-tailed t-test).

Supplementary Fig. 2

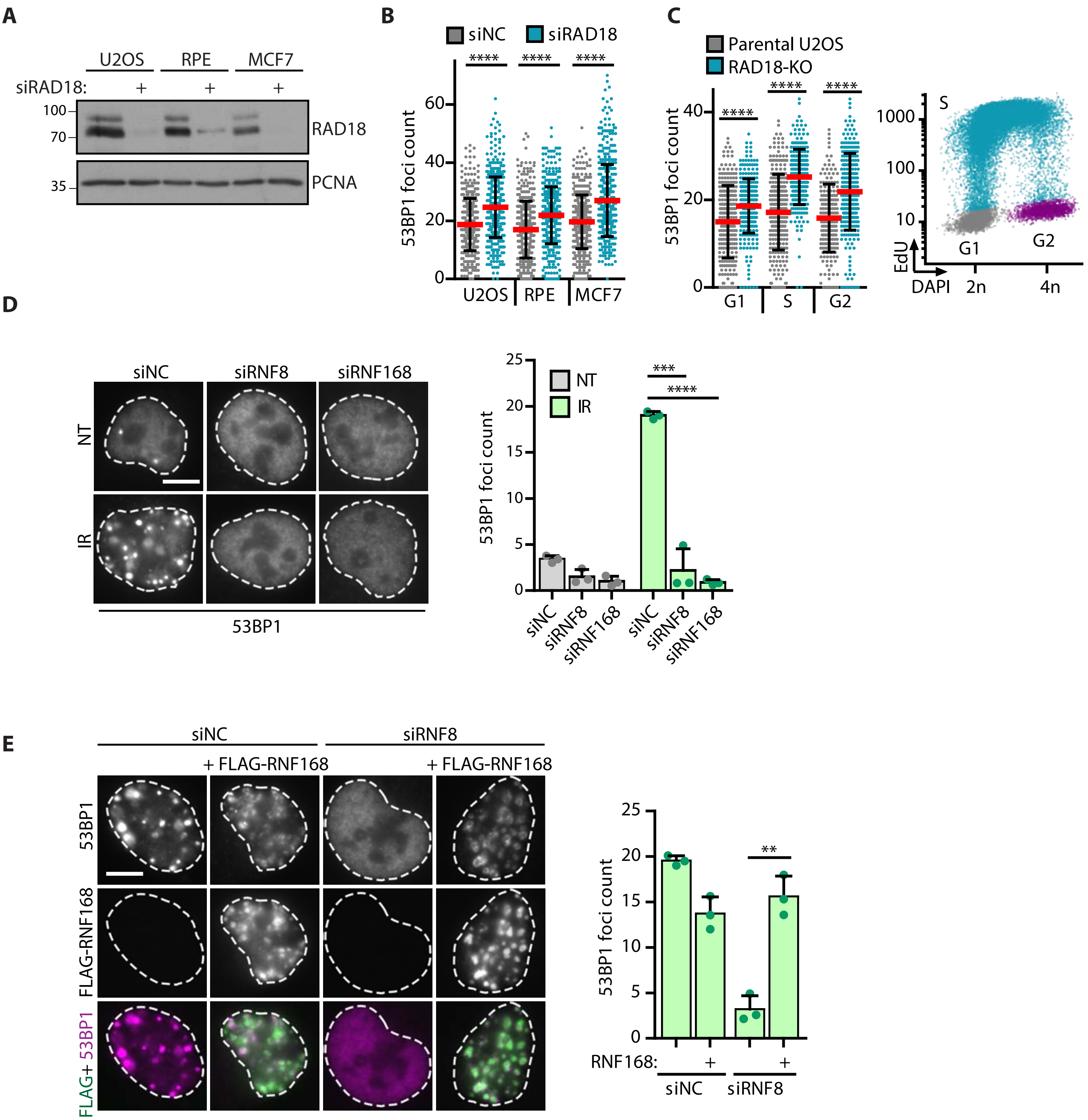

Supplementary Figure 2. RAD18 excludes 53BP1 form ubiquitinated chromatin

(A) RAD18 depletion efficiency in U2OS, RPE1 and MCF7 cells was evaluated using immunoblotting.

(B) Quantification of 53BP1 foci in U2OS, RPE1 and MCF7 cells treated with control or RAD18 siRNA for 48 h. Cells were IR-irradiated 2 h before fixation. A representative experiment from two repeats is shown (mean ±SD, n>500, Mann-Whitney test).

(C) Quantification of 53BP1 foci count in U2OS parental or RAD18-KO cells 2 h upon IR-irradiation A representative experiment from two repeats is shown (mean ±SD, n = 300, Mann-Whitney test). Right, definition of cell-cycle stages based on the total EdU and DAPI nuclear intensities.

(D) U2OS cells were transfected with control, RNF8, or RNF168 siRNA for 48 h. Where indicated, cells were IR-irradiated 2 h before fixation (scale bar 10 µm). Right, quantification of 53BP1 foci (mean +SD, n = 3, two-tailed t-test).

(E) U2OS cells were treated with control or RNF8 siRNA for 48 h and transfected with FLAG-RNF168 plasmid 19 h before IR-irradiation. Cells were fixed and stained for FLAG and 53BP1 (scale bar 10 µm). Right, quantification of 53BP1 foci in FLAG positive and negative cells is shown (mean +SD, n = 3, two-tailed t-test).

Supplementary Fig. 3

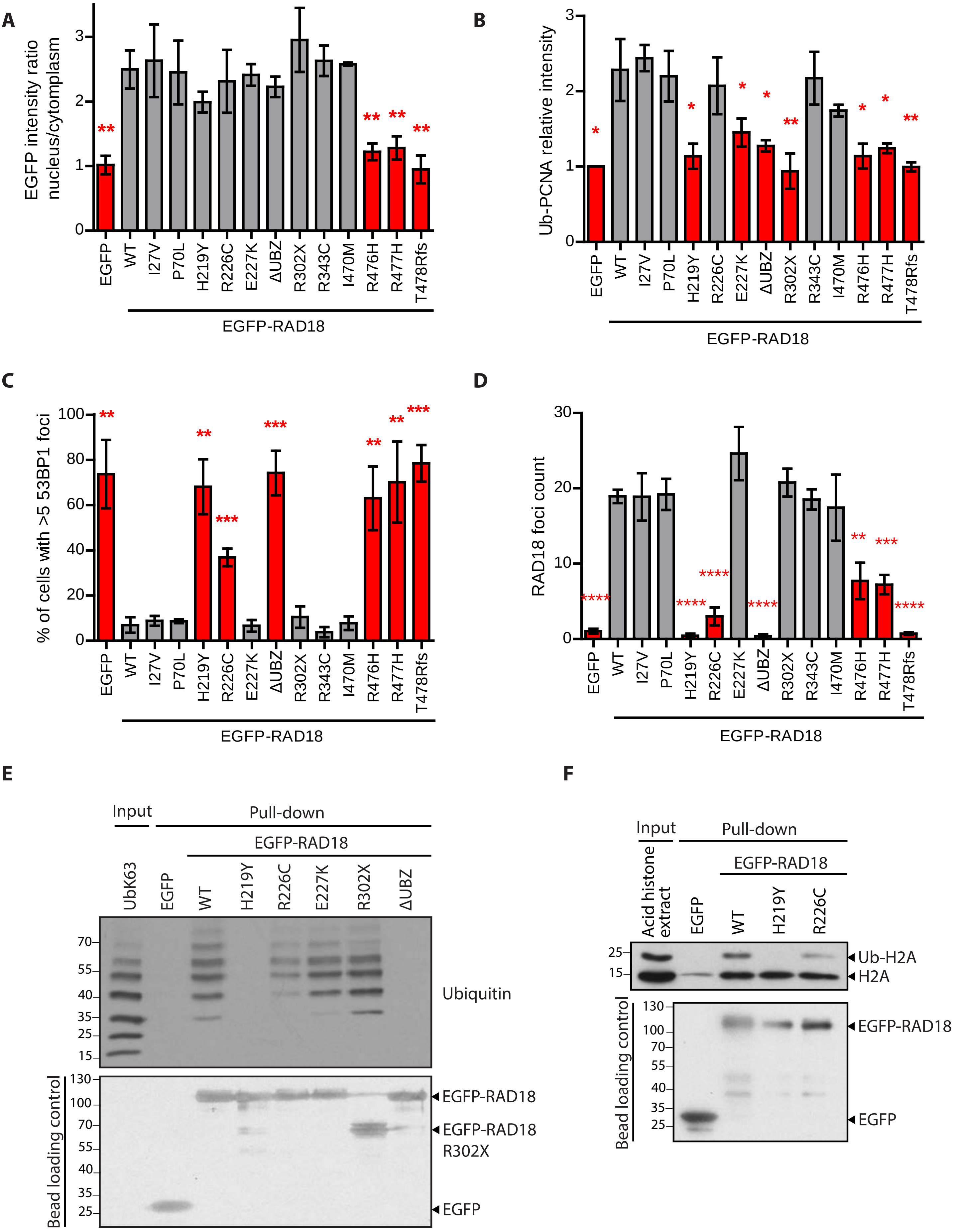

Supplementary Figure 3. Screen of RAD18 cancer variants

(A) Quantification of relative nuclear/cytoplasmic EGFP intensity in U2OS-RAD18-KO cells transfected with individual RAD18 variants. Samples significantly differing from the RAD18-WT-transfected cells are in red (mean ±SD is shown, n = 3, two-tailed t-test).

(B) Quantification of mean UbPCNA intensity in U2OS-RAD18-KO cells transfected with individual RAD18 variants. Cells were fixed 4 h after UV-irradiation. Samples significantly differing from RAD18-WT are in red (mean ±SD is shown, n = 3, two-tailed t-test).

(C) Quantification of cell fraction with more than five 53BP1 foci. U2OS-RAD18-KO cells were transfected with individual RAD18 variants and fixed 2 h after IR-irradiation. Samples significantly differing from RAD18-WT are in red (mean ±SD is shown, n = 3, two-tailed t-test).

(D) Quantification of RAD18 foci counts in U2OS-RAD18-KO cells transfected with individual RAD18 variants. Cells were fixed 2 h after IR-irradiation. Samples significantly differing from the RAD18-WT-transfected cells are in red (mean ±SD is shown, n = 3, two-tailed t-test).

(E) U2OS-RAD18-KO cells were transfected with EGFP-RAD18 variants or an EGFP empty vector control. Cells were lysed 24 h after transfection, treated with bensonase, incubated with GFP trap and extensively washed. Immunoprecipitated EGFP-RAD18 was then incubated with K63-linked polyubiquitin chains. Pulled-down polyubiquitin was analyzed using immunoblotting.

(F) U2OS-RAD18-KO cells were transfected with EGFP-RAD18 variants or an EGFP empty vector control. Cells were lysed 24 h after transfection, treated with bensonase, incubated with GFP trap and extensively washed. Immunoprecipitated EGFP-RAD18 was then incubated with acid histone extracts. Pulled-down UbH2A was analyzed using immunoblotting.

Supplementary Fig. 4

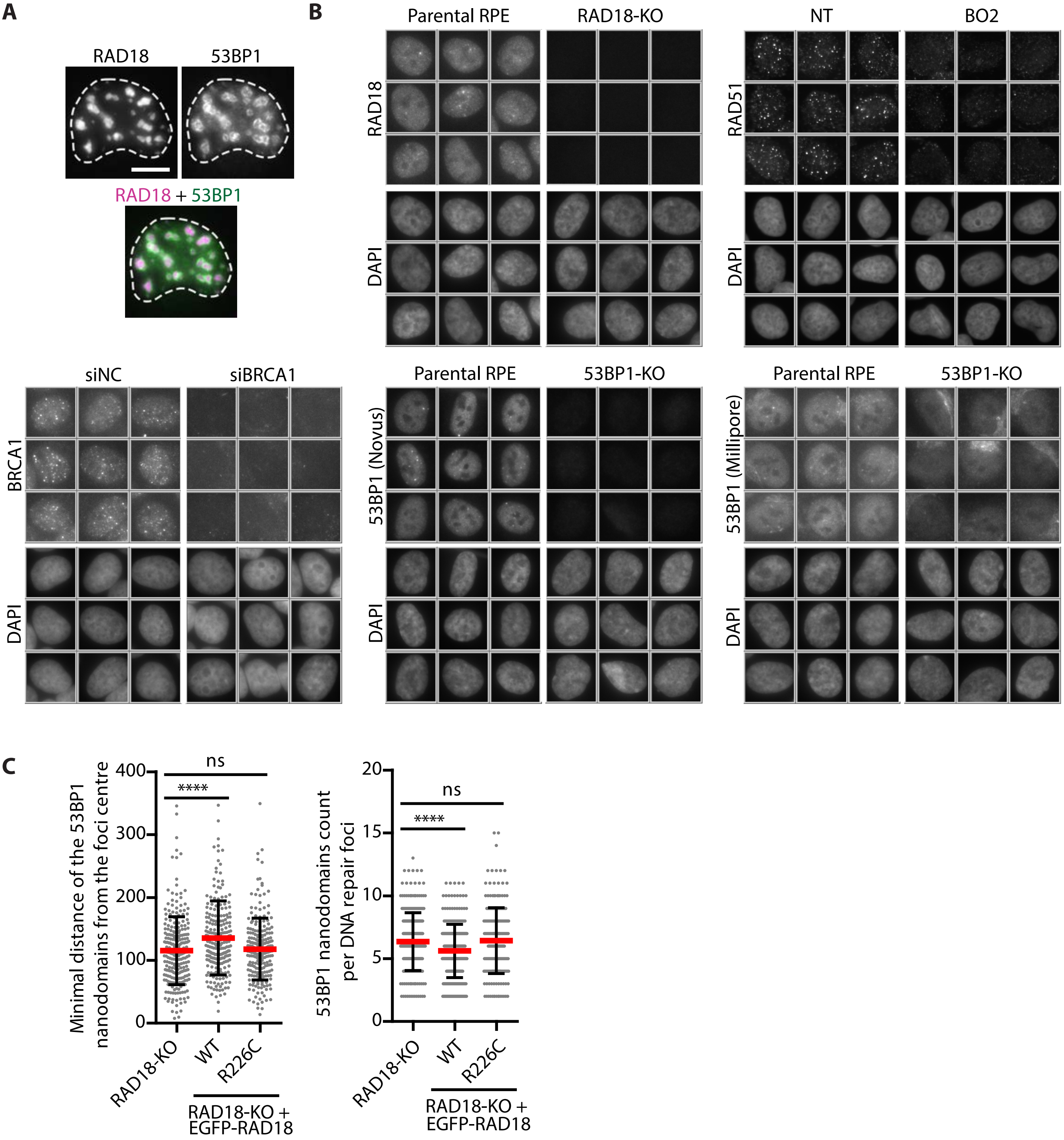

Supplementary Figure 4. Role of RAD18 in repair foci topology and antibody validation

(A) U2OS cells were transfected with FLAG-RNF168 plasmid, fixed after 24 h and stained for RAD18 and 53BP1 (scale bar 10 µm).

(B) Validation of RAD18 and 53BP1 antibodies in RPE RAD18-KO and 53BP1-KO cells. For BRCA1 antibody validation, U2OS cells were treated with BRCA1 siRNA. RAD51 antibody was validated in pre-extracted U2OS cells treated with BO2 RAD51 inhibitor prior IR-irradiation.

(C) Quantification of STED images of IR-irradiated U2OS-RAD18-KO, and RAD18-KO cells reconstituted with RAD18 WT or R226C variant. Number and the minimal distance of 53BP1 nanodomains from the foci center is shown for individual repair foci. The mean ±SD is shown, Mann-Whitney test, $n \geq 224$ foci form 20 cells from two independent experiments.

Supplementary Fig. 5

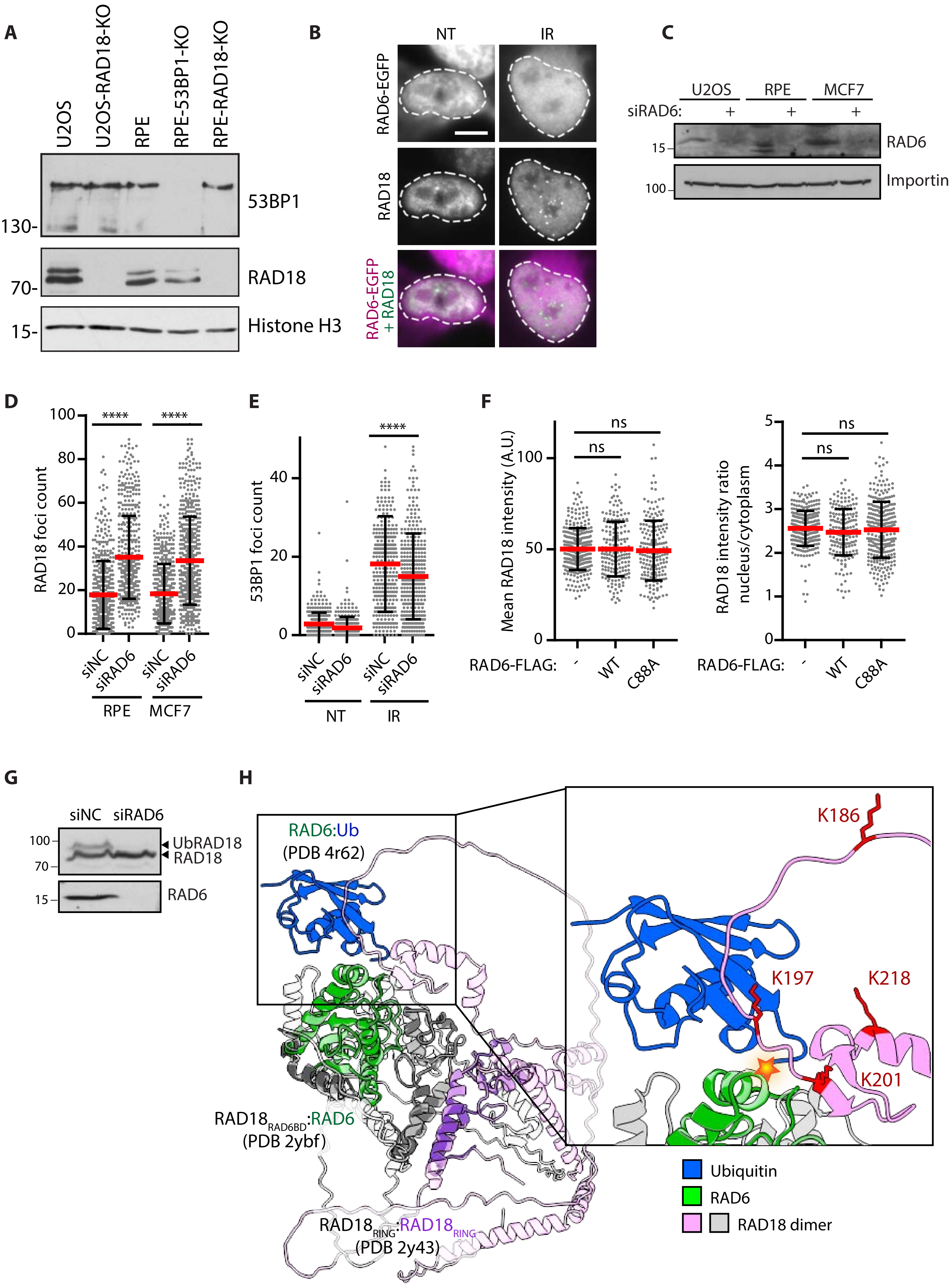

Supplementary Figure 5. Regulation of RAD18 by RAD6-mediated autoubiquitination

(A) Evaluation of 53BP1 levels in parental U2OS and RPE and RAD18-KO cells by immunoblotting. RPE-53BP1 KO cells were used as a control for antibody specificity.

(B) U2OS cells transfected with RAD6-EGFP plasmid were IR-irradiated as indicated. After 2 h, cells were stained for RAD18 (scale bar 10 µm).

(C) RAD6 depletion efficiency in U2OS, RPE1 and MCF7 cells was evaluated using immunoblotting.

(D) Quantification of RAD18 foci in RPE and MCF7 cells treated with control or RAD6 siRNA for 48 h. Cells were IR-irradiated 2 h before fixation (mean ±SD, $n \geq 500$, Mann-Whitney test).

(E) Quantification of 53BP1 foci in U2OS cells treated with control or RAD6 siRNA. Where indicated, cells were IR-irradiated 2 h before fixation (mean ±SD, $n = 350$, Mann-Whitney test).

(F) Quantification of mean RAD18 nuclear intensity (left) and nuclear/cytoplasmic mean intensity ratio (right) in U2OS cells transfected or not with RAD6-WT-FLAG wild type RAD6-C88A-FLAG (mean ±SD, $n \geq 191$, Mann-Whitney test). For D – F, representative experiments are shown from two independent repeats.

(G) Validation of the slowly migrating auto-ubiquitinated RAD18 species. Cells were harvested 48 h upon siRAD6 transfection and analyzed by immunoblotting.

(H) AlphaFold Colab was used to model interaction of RAD18 (residues 1-395) (grey) and RAD6 (green). To validate precision of the prediction, the model was superimposed with experimentally derived $RAD18_{RAD6BD}$:RAD6 structure (PDB 2ybf) (dark). The model was then aligned to another AlphaFold-modeled RAD18 molecule (residues 1-395) (pink) based on $RAD18_{RING}$:$RAD18_{RING}$ dimer structure (PDB 2y43) (dark). Finally, the model was aligned with experimentally derived RAD6:Ub structure (PDB 4r62). The star represents the thioester bond between the C-terminal glycine of ubiquitin and cysteine 88 of RAD6. The proximal lysines available for ubiquitin conjugation in the other RAD18 molecule are shown in red.

Supplementary Fig. 6

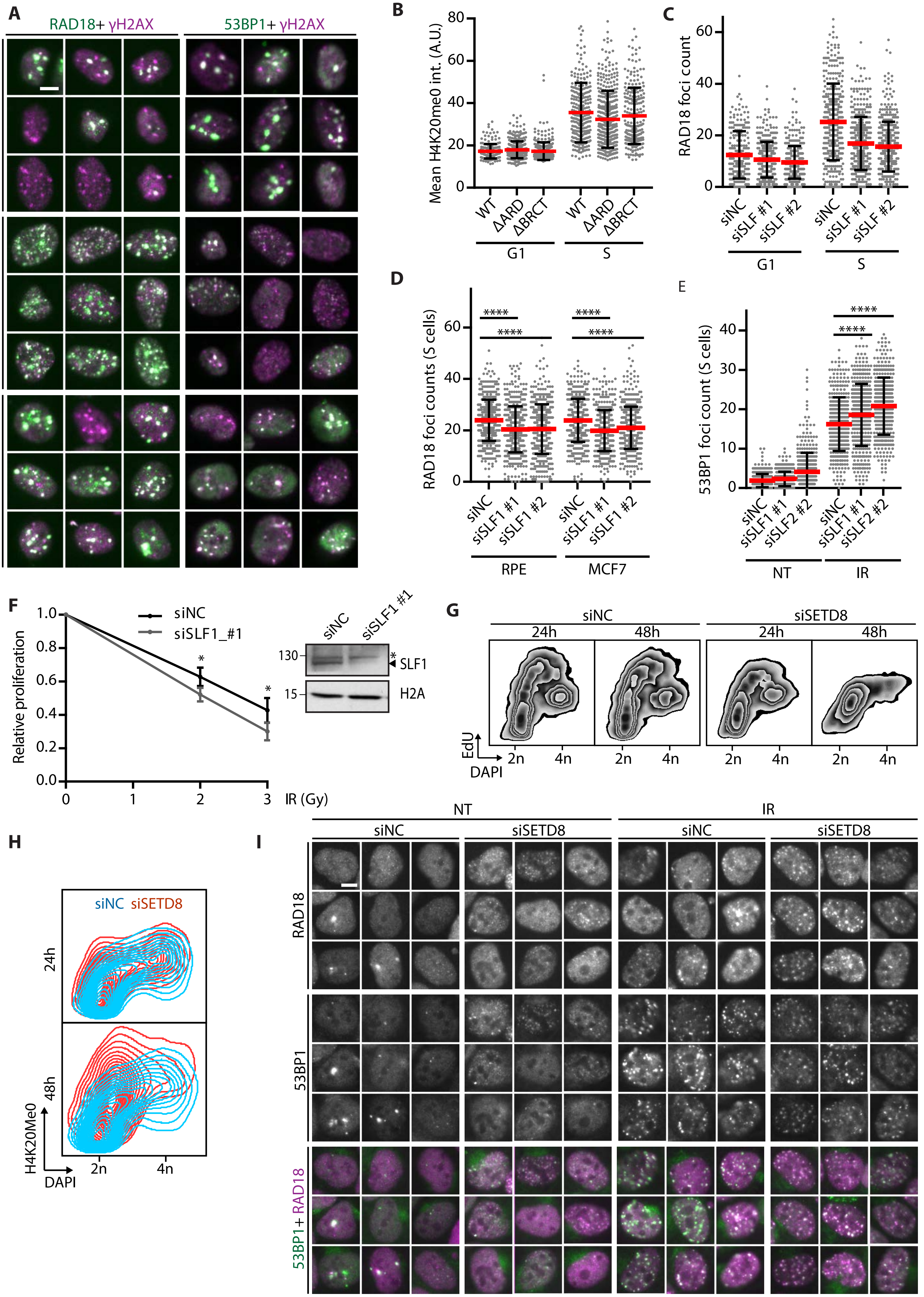

Supplementary Figure 6. RAD18 is recruited to the post-replicative chromatin

(A) RPE-iCut cells were treated overnight with Shield-1 and doxycycline to induce expression of Cas9 endonuclease that was targeted to DNA upon transfection of mixture of 12 single cutter sgRNAs. Cells were fixed 6 h post transfection, Click-iT labelled for incorporated EdU and stained for γH2AX, and 53BP1 or RAD18. Cell cycle phases were distinguished by gating on the total EdU and DAPI nuclear intensities. (scale bar 10 μm).

(B) Quantification shows mean nuclear H4K20me0 intensity in U2OS cells transfected with wild type EGFP-SLF1 or ΔBRCT and ΔARD variants. G1 and S cell cycle phases were distinguished by gating on the total EdU and DAPI nuclear intensities (mean ±SD, n ≥ 203). A representative experiment is shown from two independent repeats.

(C) Quantification of RAD18 foci count in U2OS cells treated with control or two independent SLF1 siRNAs for 48 h. Cells were IR-irradiated 2h before fixation. G1 and S cell cycle phases were distinguished by gating on the total EdU and DAPI nuclear intensities (mean ±SD, n ≥ 226). A representative experiment is shown from two independent repeats.

(D) Quantification of RAD18 foci count in EdU positive RPE1 and MCF7 cells treated with control or two different SLF1 siRNAs for 48 h. Cells were IR-irradiated 2 h before fixation (mean ±SD, n ≥ 501, Mann-Whitney test).

(E) Quantification of 53BP1 foci count in EdU positive U2OS cells treated with control or two different SLF1 siRNAs for 48 h. Cells were IR-irradiated 2 h before fixation (mean ±SD, n = 400, Mann-Whitney test).

(F) Relative proliferation of U2OS cells treated with control and SLF1 #1 siRNA was evaluated using resazurin viability assay 7 days after irradiation with indicated doses IR (mean +SD is shown, n = 3, two-tailed t-test). Right, evaluation of depletion efficiency by immunoblotting. The asterisk indicates a non-specific band.

(G) High-content microscopy analysis of cell cycle phase distribution in U2OS cells treated with control or SETD8 siRNA for 24 h or 48 h. Plotted are the total EdU and DAPI nuclear intensities.

(H) High-content microscopy quantification of H4K20me0 in U2OS cells treated with control or SETD8 siRNA for 24 h or 48 h. Plotted are the mean H4K20me0 and total DAPI nuclear intensities.

(I) U2OS cells treated with control or SETD8 siRNA for 24 h were IR-irradiated as indicated, fixed after 2 h and stained for RAD18 and 53BP1 (scale bar 10 μm).

Supplementary Fig. 7

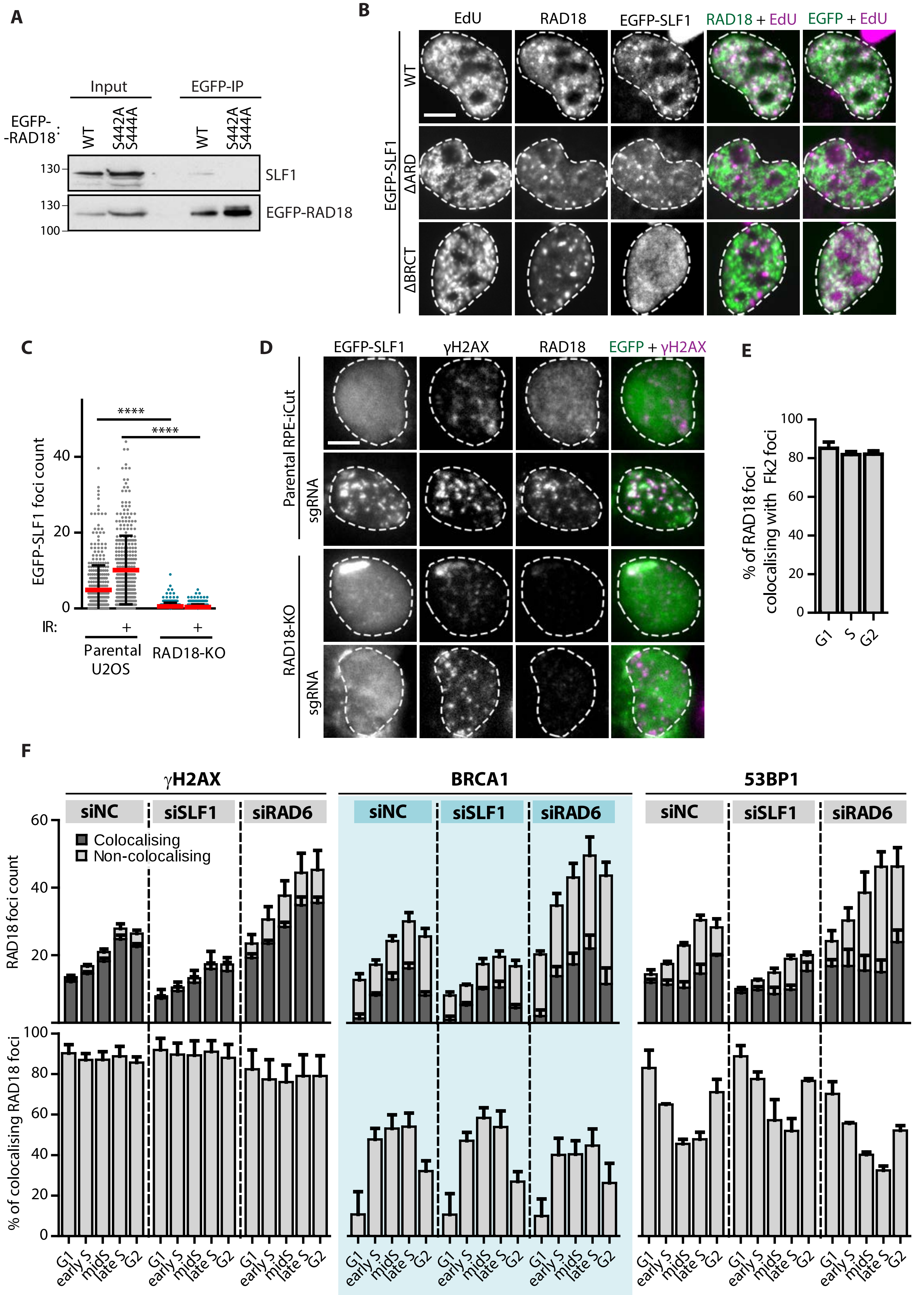

Supplementary Figure 7. RAD18 is recruited to DNA lesions with SLF1

(A) Co-immunoprecipitation of SLF1 from U2OS cells expressing EGFP-RAD18-WT and S442A/S444A variant. Cells were harvested, treated with bensonase and incubated with GFP trap. Co-precipitated endogenous SLF1 was analyzed using immunoblotting.

(B) Colocalization of RAD18 and EGFP with EdU in early S cells transfected with EGFP-SLF1 variants. Cells were IR-irradiated 24 h upon transfection, pre-extracted, fixed, Click-iT-labelled for incorporated EdU and stained for RAD18 (scale bar 10 µm).

(C) Quantification of EGFP-SLF1 foci in parental U2OS and RAD18-KO cells transfected with EGFP-SLF1. Where indicated, cells were IR-irradiated and fixed after 2 h (mean ±SD, n ≥ 354, Mann-Whitney test). A representative experiment form two independent repeats is shown.

(D) Colocalization of EGFP-SLF1 and RAD18 with γH2AX at DSBs induced in RPE-iCut cells. RPE-iCut cells were treated overnight with Shield-1 and doxycycline to induce expression of Cas9 endonuclease that was targeted to DNA upon transfection of mixture of 12 single cutter sgRNAs. Cells were pre-extracted 6 h after transfection, fixed, and stained for γH2AX and RAD18 (scale bar 10 µm).

(E) Percentage of RAD18 foci colocalizing with FK2 foci in U2OS cells IR-irradiated with 3 Gy. Cells were pulse-labeled with EdU prior irradiation. After 4 h, cells were pre-extracted, fixed, Click-iT-labelled for incorporated EdU and stained with RAD18 and FK2 antibodies (mean +SD, n = 3).

(F) Count (count) and percentage (bottom) of RAD18 foci colocalizing with γH2AX, BRCA1, and 53BP1 foci, respectively. U2OS cells were pulse-labeled with EdU prior irradiation with 3 Gy. After 4 h or 6 h (for BRCA1), cells were pre-extracted, fixed, Click-iT-labelled for incorporated EdU and stained with RAD18 and γH2AX, BRCA1, and 53BP1 antibody, respectively (mean +SD, n = 3).

Supplementary Fig. 8

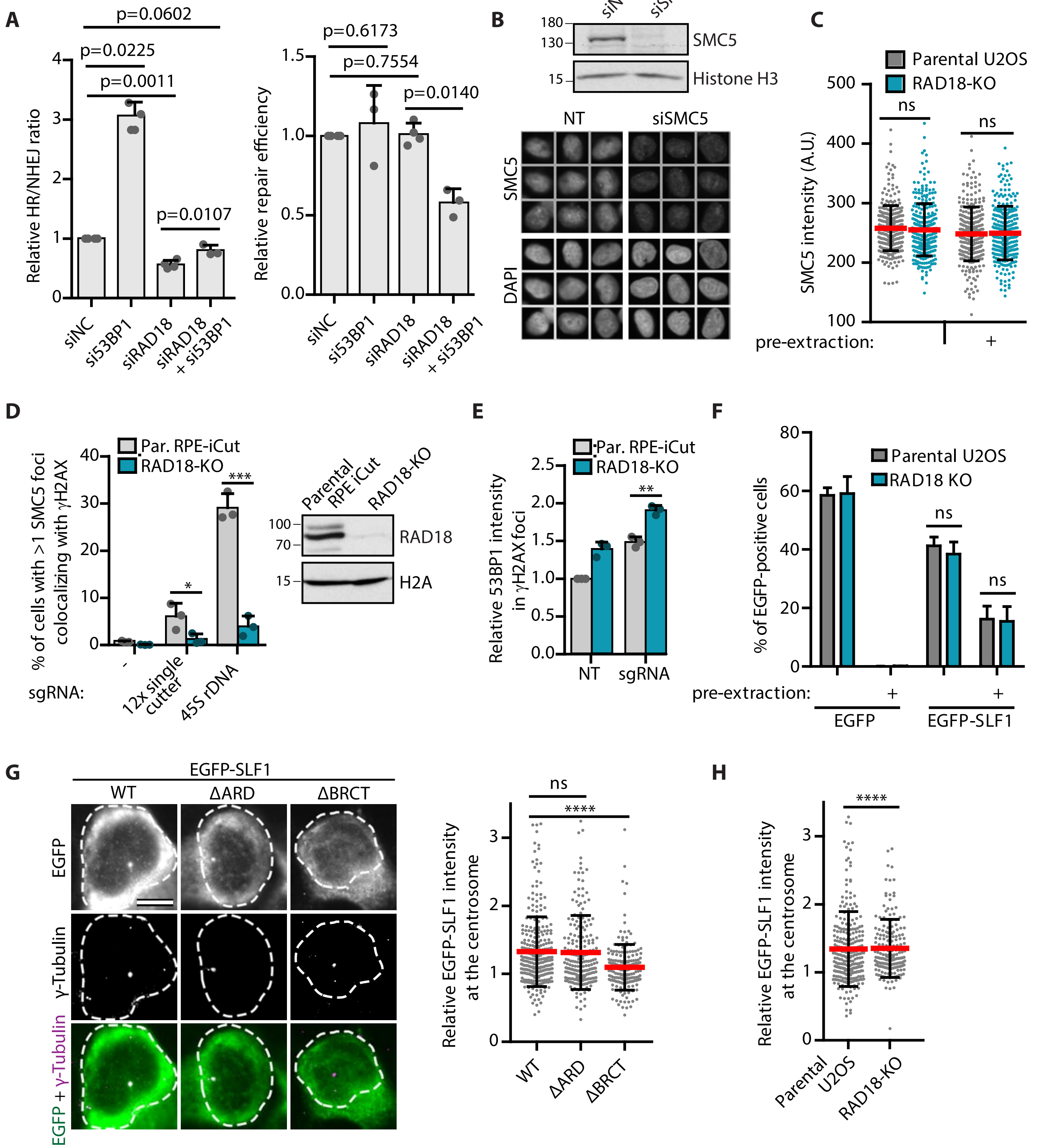

Supplementary Figure 8. RAD18 recruits SMC5 to DSBs

(A) HR/NHEJ repair ratio (left) and the total repair efficiency (right) both normalized to control in traffic light reporter U2OS cells treated with indicated siRNAs (mean +SD, n ≥ 3, one-sample two-tailed t-test for comparison with control, otherwise two-sample two-tailed t-test).

(B) SMC5 depletion efficiency in U2OS cells treated with control or SMC5 siRNA was evaluated using immunoblotting and immunofluorescence.

(C) Quantification of the mean nuclear SMC5 intensity in parental U2OS and RAD18-KO cells. Where indicated, cells were pre-extracted prior fixation (mean ±SD, n = 300, Mann-Whitney test).

(D) Parental RPE-iCut cells and RAD18-KO were treated overnight with Shield-1 and doxycycline to induce expression of Cas9 endonuclease that was targeted to DNA upon transfection of sgRNA targeting 45S rDNA repeats or a mixture of 12 single cutter sgRNAs. Cells were pre-extracted 6 h after transfection, fixed, and stained for γH2AX and SMC5. Quantification of cell fraction with > 1 SMC5 foci colocalizing with γH2AX foci (mean +SD, n = 3, two-tailed t-test). Right, validation of RPE-iCut-RAD18-KO cells by immunoblotting.

(E) Parental RPE-iCut cells and RAD18-KO were treated overnight with Shield-1 and doxycycline to induce expression of Cas9 and then transfected with a mixture of 12 single cutter sgRNAs. Cells were pre-extracted 6 h after transfection, fixed, and stained for γH2AX and 53BP1 (scale bar 10 µm). Relative mean 53BP1 intensity in γH2AX foci is normalized to non-treated parental cells (mean +SD, n = 3, two-tailed t-test).

(F) Percentage of EGFP positive cells in parental U2OS and RAD18-KO cells transfected with EGFP-SLF1 or an empty EGFP vector and pre-extracted or not as indicated (mean is shown +SD, n = 3, two-tailed t-test)

(G) U2OS cells were transfected with EGFP-SLF1 or ΔBRCT and ΔARD variants, methanol fixed, and stained for γTubulin (scale bar 10 µm). Right, quantification of relative EGFP-SLF1 intensity at the γTubulin-stained centrosome (mean ±SD, n ≥ 200, Mann-Whitney test).

(H) Quantification of relative EGFP-SLF1 intensity at the γTubulin-stained centrosome in parental U2OS cells and RAD18-KO cells transfected with EGFP-SLF1 (mean ±SD, n ≥ 196, Mann-Whitney test).
